# All-trans retinoic acid enhances, and a pan-RAR antagonist counteracts, the stem cell promoting activity of *EVI1* in acute myeloid leukemia

**DOI:** 10.1038/s41419-019-2172-2

**Published:** 2019-12-10

**Authors:** Chi Huu Nguyen, Katharina Bauer, Hubert Hackl, Angela Schlerka, Elisabeth Koller, Anastasiya Hladik, Dagmar Stoiber, Johannes Zuber, Philipp B. Staber, Andrea Hoelbl-Kovacic, Louise E. Purton, Florian Grebien, Rotraud Wieser

**Affiliations:** 1 Division of Oncology, Clinic of Medicine I, Medical University of Vienna, Vienna, Austria; 2 Comprehensive Cancer Center, Vienna, Austria; 3 Division of Bioinformatics, Biocenter, Medical University of Innsbruck, Innsbruck, Austria; 4 Medical Department for Leukemia Research and Hematology, Hanusch Hospital, Vienna, Austria; 5 Research Laboratory of Infection Biology, Clinic of Medicine I, Medical University of Vienna, Vienna, Austria; 6 Ludwig Boltzmann Institute for Cancer Research, Vienna, Austria; 7 Institute of Pharmacology, Medical University of Vienna, Vienna, Austria; 8 Institute of Molecular Pathology, Vienna, Austria; 9 Division of Hematology and Hemostaseology, Clinic of Medicine I, Medical University of Vienna, Vienna, Austria; 10 Institute of Pharmacology and Toxicology, University of Veterinary Medicine, Vienna, Austria; 11 Stem Cell Regulation Unit, St. Vincent’s Institute of Medical Research and Department of Medicine at St. Vincent’s Hospital, The University of Melbourne, Melbourne, Australia; 12 Institute of Medical Biochemistry, University of Veterinary Medicine, Vienna, Austria

**Keywords:** Cancer stem cells, Haematological cancer

## Abstract

*Ecotropic virus integration site 1* (*EVI1*), whose overexpression characterizes a particularly aggressive subtype of acute myeloid leukemia (AML), enhanced anti-leukemic activities of all-trans retinoic acid (atRA) in cell lines and patient samples. However, the drivers of leukemia formation, therapy resistance, and relapse are leukemic stem cells (LSCs), whose properties were hardly reflected in these experimental setups. The present study was designed to address the effects of, and interactions between, *EVI1* and retinoids in AML LSCs. We report that *Evi1* reduced the maturation of leukemic cells and promoted the abundance, quiescence, and activity of LSCs in an *MLL-AF9*-driven mouse model of AML. atRA further augmented these effects in an *Evi1* dependent manner. EVI1 also strongly enhanced atRA regulated gene transcription in LSC enriched cells. One of their jointly regulated targets, *Notch4*, was an important mediator of their effects on leukemic stemness. In vitro exposure of leukemic cells to a pan-RAR antagonist caused effects opposite to those of atRA. In vivo antagonist treatment delayed leukemogenesis and reduced LSC abundance, quiescence, and activity in Evi1^high^ AML. Key results were confirmed in human myeloid cell lines retaining some stem cell characteristics as well as in primary human AML samples. In summary, our study is the first to report the importance of *EVI1* for key properties of AML LSCs. Furthermore, it shows that atRA enhances, and a pan-RAR antagonist counteracts, the effects of *EVI1* on AML stemness, thus raising the possibility of using RAR antagonists in the therapy of EVI1^high^ AML.

## Introduction

Acute myeloid leukemia (AML) is organized in a hierarchical manner, i.e., the bulk of the leukemic cell mass is derived from mostly quiescent leukemic stem cells (LSCs), which are the source of disease emergence, therapy resistance, and relapse^1^. AML is caused by specific cytogenetic aberrations, point mutations, and epigenetic and transcriptional changes^2,3^, which occur in hematopoietic stem cells (HSCs) or progenitor cells, causing their transformation into LSCs^1,4,5^. One such change is overexpression of *EVI1*, which is present in around 10% of patients and associated with a dismal prognosis^6–8^. *EVI1* is located in chromosome band 3q26 and codes for a zinc finger transcription factor^9,10^. In normal hematopoiesis, *EVI1* is highly expressed in immature cells, but rapidly down-regulated during differentiation^11,12^; accordingly, it promoted the abundance, quiescence, and activity of murine HSCs^11,13^. Experimental expression or knock-down of *Evi1* in mouse models of AML promoted or delayed myeloid leukemogenesis, respectively^12–15^, and activation of *EVI1* through vector integration caused AML in a gene therapy trial for chronic granulomatous disease^16^. The most prominent causes of *EVI1* overexpression in human AML are juxtaposition to a strong enhancer, or transcriptional induction by leukemia-associated fusion proteins. Specifically, *EVI1* is brought under control of the *GATA2* enhancer in cases with inv(3)(q21q26) or t(3;3)(q21;q26)^17,18^, and up-regulated through direct promoter binding by lysine methyltransferase 2A (KMT2A; more commonly known as mixed lineage leukemia, MLL) fusion proteins, which result from 11q23 rearrangements^12,19^. MLL fusion proteins transformed both murine HSCs and progenitor cells, but enhanced *Evi1* expression only in the former. This suggested that the presence or absence of *EVI1* overexpression, each observed in about half of the patients, reflects the cell type in which the transforming event occurred also in human *MLL* rearranged AML^4,7,12,19,20^.

All-trans retinoic acid (atRA) acts through nuclear receptor transcription factors composed of a retinoic acid receptor (RAR) and a retinoid X receptor (RXR) subunit, and promotes both normal granulocytic differentiation and the abundance, quiescence, and activity of HSCs^21–24^. Furthermore, atRA is highly effective as a therapeutic agent in acute promyelocytic leukemia (APL), a subtype of AML characterized by fusion proteins involving RARα. The most frequent of these, PML-RARα, does not respond to physiological doses of atRA, yet myeloid differentiation is restored by pharmacological levels of this agent^25^. In contrast, even though atRA also promoted the differentiation of non-APL AML blasts, clinical trials have failed to reveal any clear therapeutic benefit in these patients^26–28^. Certain molecularly or genetically defined subgroups of non-APL AML were suggested to gain a survival advantage from atRA, but no consistent picture has yet emerged^26,27,29,30^.

atRA regulated expression of *EVI1* both in cell lines and in primary AML cells^30,31^. Conversely, EVI1 acted as a modulator of transcriptional responses to atRA, and augmented anti-leukemic activities of atRA in human myeloid cell lines and primary AML cells^29,30^. However, since AML is a stem cell-driven disease, it is important to understand the impact of potential therapeutics on LSCs. Relatively little is known about the effects of atRA on, and even less about the role of *EVI1* in, AML LSCs. Here, we report that *EVI1* promoted essential properties of LSCs, and atRA enhanced its effects. Furthermore, EVI1 strongly augmented atRA regulated gene transcription in LSC enriched cells, and one of their joint targets, *Notch4*, was a relevant mediator of their effects on leukemic stemness. Conversely, a pan-RAR antagonist reduced AML stemness and delayed leukemogenesis, raising the possibility of using RAR antagonists in the therapy of EVI1^high^ AML.

## Methods

### Ethics statement

Animal experiments were approved by the Animal Ethics Committee of the Medical University of Vienna and the Austrian Federal Ministry of Education, Science, and Research (GZ66.009/0308-WF/V/3b/2015). Federation of European Laboratory Animal Science Associations guidelines to minimize animal distress and suffering were followed. Experiments with primary AML samples were approved by the Ethics Committee of the Medical University of Vienna (EK 1394/2019) and conducted in accordance with the declaration of Helsinki.

### Generation of *MA9*-driven murine AML with high or low expression of *Evi1*

HSC enriched Lin^−^ Sca-1^+^ c-Kit^+^ (LSK) cells and common myeloid progenitors (CMPs; Lin^−^ Sca-1^−^ c-Kit^+^ CD34^+^ CD16/CD32^low^ cells; Supplementary Fig. S1A) were isolated from bone marrow (BM) of 6–8 week old C57BL/6 mice (Department of Laboratory Animal Science & Genetics, Himberg, Austria), transduced with pMSCV_MA9_IRES_Venus, and transplanted into sub-lethally irradiated congenic recipient mice^4,32^. Venus^+^ BM or spleen cells from terminally ill mice were considered leukemic cells (LCs), and are referred to as LC^LSK_MA9^ and LC^CMP_MA9^, respectively. To knock down *Evi1* in LC^LSK_MA9^, they were transduced with lentiviral vectors (pLKO.1_puro_CMV_TagRFP) containing shEvi1_41, shEvi1_43, shEvi1_44 or control shRNA SHC012 (shCtrl; Sigma-Aldrich). Venus^+^ RFP^+^ cells were used for transplantation. Venus^+^ RFP^+^ cells from BM or spleen of terminally ill recipient mice were designated LC^LSK_MA9_shEvi1^ and LC^LSK_MA9_shCtrl^, respectively.

### Ex vivo culture and flow cytometric analyses of cells from leukemic mice

BM cells from leukemic mice were cultured in IMDM medium containing 10% FBS, 1% l-Glutamine (all from Thermo Fisher Scientific), 50 ng/ml mSCF, 10 ng/ml mIL-3, 10 ng/ml mTPO, 10 ng/ml mFlt3L (all from Peprotech), and 10 ng/ml mIL-6 (Biolegend). For treatment, cells were seeded at a density of 2 × 10^5^ per ml and incubated with 1 µM atRA (Sigma-Aldrich), 1 µM pan-RAR antagonist AGN193109 (Tocris), 5 µM γ-secretase inhibitor DAPT (Stem Cell Technologies), or the corresponding amounts of DMSO (Sigma-Aldrich) for 72 h, unless indicated otherwise. By gating on fluorescence marker positive cells, all analyses were restricted to LCs. LC differentiation and the proportion of LSC enriched cells (LSCe; Venus^+^ or Venus^+^ RFP^+^, Lin^−^ Sca1^−^ c-Kit^+^ CD34^+^ CD16/CD32^hi^ cells^4,5^) were determined by flow cytometric analysis of BM cells stained with the respective antibodies (Supplementary Table S1).

To determine the cell cycle distribution of LSCe, BM cells were stained for LSCe surface markers, fixed and permeabilised in Cytofix/Cytoperm (BD Biosciences), stained with Ki-67 antibody (Supplementary Table S1) and DAPI (Sigma-Aldrich), and subjected to flow cytometry. The cut-off for Ki-67 positivity was determined using an isotype control antibody. Among cells in the LSCe gate, Ki-67^-^ cells with a 2N DNA content were considered to be in G_0_. Flow cytometry was performed on an LSR Fortessa SORP (BD Biosciences), and data were analyzed with FlowJoX software (Treestar).

### Serial replating assay

For serial replating assays, BM LCs were incubated with 1 µM atRA, 5 µM DAPT, or the corresponding amounts of DMSO for 72 h, or left untreated when obtained from antagonist treated mice. 2000 cells per condition were seeded into methyl cellulose (MethoCult GF M3434; Stem Cell Technologies). Colonies were counted after 7 days, and 2000 cells per condition were used for replating.

### RNA sequencing (RNA-seq) and bioinformatics analyses

LSCe^LSK_MA9_shCtrl^ (from three different mice) and LSCe^LSK_MA9_shEvi1^ (shEvi1_41, shEvi1_43, and shEvi1_44) were isolated from spleens of terminally ill mice, recovered for 24 h, and incubated with 1 µM atRA or the corresponding amount of DMSO for another 24 h. RNA isolation, library preparation, RNA-seq, and data analysis are described in Supplementary Methods. By applying a false discovery rate (FDR) of <0.05, genes regulated by EVI1 in the absence (Er_D) or presence (Er_A) of atRA, and genes regulated by atRA in the absence (Ar_shE) or presence (Ar_shC) of EVI1 were identified. Furthermore, genes were identified whose expression patterns mirrored the observed biological effects, i.e., which showed little or no regulation by atRA in shEvi1 cells, and whose regulation by EVI1 was enhanced by atRA (Er_D/Ar_shC; see Supplementary Methods for detailed definition). Heatmaps were generated based on z-score transformation of mean normalized counts across all four conditions. RNA-seq data were deposited in the Gene Expression Omnibus (GSE123255).

### In vivo limiting dilution assay

BM LC^LSK_MA9_shCtrl^ and LC^LSK_MA9_shEvi1_44^ were incubated with 1 µM atRA or the corresponding amount of DMSO. After 72 h, 5000, 2000, 750, 250, 125, and 25 cells were transplanted into sub-lethally irradiated 6–8 week old female C57BL/6 recipient mice (5 mice per condition). Two independent experiments were performed, and data were combined for the final analysis. Mice were sacrificed when signs of disease became evident, or at the end of the experiment (4 months after transplantation), and BM cells were collected for flow cytometric analysis. Recipient mice with less than 1% Venus^+^ RFP^+^ cells in BM were considered as non-responders. LSC frequency was calculated by applying the maximum likelihood method using ELDA software^33^.

### In vivo treatment with the pan-RAR antagonist AGN193109 and secondary transplantation

Sub-lethally irradiated C57BL/6J mice were transplanted with 40,000 LC^LSK_MA9_shCtrl^. Seven days post transplantation (at which time Venus^+^ RFP^+^ cells started to appear in PB), mice were randomly divided into two groups (*n* = 4/group) and treated daily with either AGN193109 (1 mg/kg) or an equivalent amount of vehicle (2.55% DMSO in PBS) by intraperitoneal injection for 14 days^24,34,35^. Terminally ill mice were sacrificed, and the proportion, cell cycle distribution, and activity of LSCe/LSCs in BM were determined as described above. For secondary transplantations, sub-lethally irradiated C57BL/6J mice were transplanted with 20,000 BM LCs derived from terminally ill, AGN193109 or vehicle treated mice (*n* = 5/group).

### Knock-down of *EVI1* in human myeloid cell lines

Culture of HNT-34 and UCSD/AML1 cells, their transduction with shEVI1_1, shEVI1_2 (Open Biosystems), and control shRen in LT3REVIR^36^, as well as atRA treatment and biological assays are described in Supplementary Methods.

### Primary AML samples

Cryopreserved primary AML samples were provided by the Hanusch hospital (Vienna, Austria). They were thawed in a 25 °C water bath and washed with RPMI medium containing 5 µg/ml DNAse (Sigma-Aldrich). Cells were incubated for 60 min at 37 °C and 5% CO_2_ with RPMI medium containing 50 µg/ml DNAse to prevent cell clumping, washed with PBS, and cultured in RPMI medium supplemented with 10% FBS, 1% glutamine, 1% Penicillin/Streptomycin, and 100 ng/ml each of SCF, IL3, and G-CSF (all from Peprotech). For treatment, cells were seeded at a density of 2 × 10^5^/ml and incubated with 1 µM atRA, 1 µM AGN193109, or the corresponding amount of DMSO for 72 h. At the end of the incubation time, cells were harvested, stained with the respective antibodies (Supplementary Table S1), and subjected to flow cytometric analysis of CD11b expression or of the cell cycle distribution of the LSC enriched CD34^+^ CD38^−^ population. Another portion of cells was transferred to methyl cellulose (MethoCult H4434, Stemcell Technologies) at a concentration of 1 × 10^5^ cells per well of a 6-well plate. Technical duplicates were performed, and total colonies were counted after 14 days.

### Statistical analyses

For each experiment, independent biological replicates were performed; their numbers are indicated in the figure legends. In the case of ex vivo experiments with murine LCs, cells for these replicates were obtained from different mice. Bar graphs depict means±SEM. Significance of differences between two independent groups was calculated using two-sided Student’s *t*-test; significance of differences between multiple groups was determined by two-way ANOVA followed by Bonferroni’s post-hoc test. The log-rank test was used to evaluate survival differences between groups of mice. Analyses were performed using GraphPad Prism 6 software (GraphPad Software, San Diego, CA, USA). LSC frequencies were calculated by applying the maximum likelihood method using ELDA software and statistical significance of their differences was assessed using the Chi-square test. *p*-values <0.05 were considered statistically significant.

### Additional methods

Additional and more detailed methods are described in Supplementary Methods.

## Results

### Evi1^high^ LC^LSK_MA9^ exhibit a higher degree of stemness than Evi1^low^ LC^CMP-MA9^, and atRA further enhances LSC properties only in the former

**Fig. 1:**
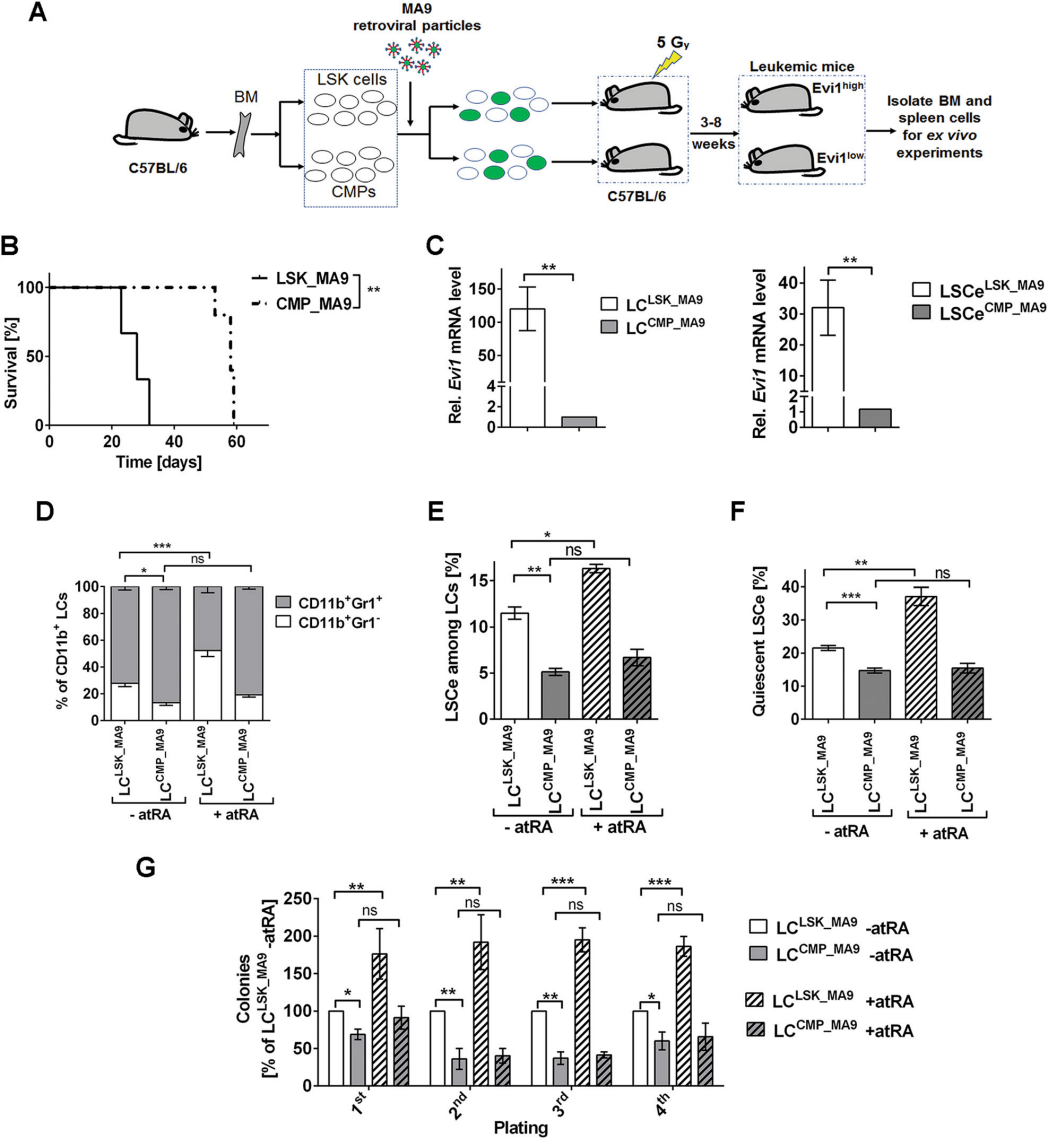
Differential effects of atRA on maturation and stemness of Evi1^high^ LC^LSK_MA9^ and Evi1^low^ LC^CMP_MA9^. **a** Schematic of experimental design. BM, bone marrow. **b** Kaplan–Meier plot of mice transplanted with *MA9* transduced LSK cells and CMPs (300,000 cells/mouse). *n* = 3/group; **p < 0.01; log-rank test. **c** Relative *Evi1* mRNA levels in BM LC^LSK_MA9^ and LC^CMP_MA9^ (left panel) and the corresponding LSCe (right panel). qRT-PCR; *n* = 3; **p < 0.01; *t*-test. **d**–**g** BM cells (**d**–**f**) or BM LCs (Venus^+^ cells; panel **g**) from terminally ill mice were treated with 1 µM atRA or solvent for 3 days. *n* = 3; *p < 0.05; **p < 0.01; ***p < 0.001; ns, not significant; ANOVA followed by Bonferroni's post-hoc test. **d** Myeloid differentiation. **e** Proportions of LSCe among LCs. **f** Proportions of quiescent LSCe (LSCe in G_0_). **g** Colony formation in methyl cellulose, presented as percent of solvent-treated LC^LSK_MA9^ in each round of plating.

To investigate the effects of *Evi1* and atRA on AML stemness, an *MA9-*driven mouse model was used (Fig. 1a). In agreement with previous reports^4,37^, both LSK cells and CMPs transduced with pMSCV_MA9_IRES_Venus caused AML-like disease upon transplantation into sub-lethally irradiated mice, but LSK-derived disease displayed shorter disease latencies and higher white blood cell counts (WBC) (Fig. 1b; Supplementary Fig. S1B, C). Also as shown before^12,19,20^, *Evi1* expression levels were substantially (>100-fold) higher in LC^LSK_MA9^ than in LC^CMP_MA9^ (Fig. 1c). To determine the impact of the cell of origin, and of atRA, on LC maturity, LC^LSK_MA9^ and LC^CMP_MA9^ were treated with atRA or solvent and subjected to flow cytometry for the myeloid differentiation markers Gr-1 and CD11b^38^. The proportion of immature (Gr-1^−^) among myeloid (CD11b^+^) cells was higher for LC^LSK_MA9^ than for LC^CMP_MA9^. Treatment with atRA for three or seven days significantly enhanced the frequency of immature cells in LC^LSK_MA9^, but had only a small, non-significant effect in LC^CMP_MA9^ (Fig. 1d; Supplementary Fig. S1D, E).

In *MA9*-driven murine AML, LSCs are strongly enriched in a cell population carrying the immuno-phenotype of granulocyte macrophage progenitors^4,5^. We therefore refer to fluorescence marker positive cells with this immuno-phenotype as LSCe (LSCe^LSK_MA9^, LSCe^CMP_MA9^). Like in bulk LCs, the expression of *Evi1* was substantially higher in LSCe^LSK_MA9^ than in LSCe^CMP_MA9^ (Fig. 1c). In agreement with their derivation from a stem cell enriched population, LC^LSK_MA9^ contained a higher proportion of LSCe than LC^CMP_MA9^. Also, atRA increased LSCe abundance among LC^LSK_MA9^, but had a smaller, non-significant effect in LC^CMP_MA9^ (Fig. 1e; Supplementary Fig. S1F, G). Moreover, a higher proportion of LSCe^LSK_MA9^ than of LSCe^CMP_MA9^ accumulated in the G_0_ phase of the cell cycle, and atRA further enhanced the proportion of quiescent LSCe^LSK_MA9^, but not LSCe^CMP_MA9^ (Fig. 1f; Supplementary Fig. S1H). In a serial replating assay, which reflects stem cell activity, LC^LSK_MA9^ displayed higher colony formation potential than LC^CMP_MA9^, and atRA increased clonogenic activity of LC^LSK_MA9^, but not LC^CMP_MA9^ (Fig. 1g). In summary, our data demonstrate that atRA prevented LC maturation, and promoted LSC properties more efficiently in Evi1^high^ LC^LSK_MA9^ than in Evi1^low^ LC^CMP_MA9^.

### *Evi1* increases stemness, and facilitates further augmentation of stemness by atRA, in *MA9*-driven murine AML

**Fig. 2:**
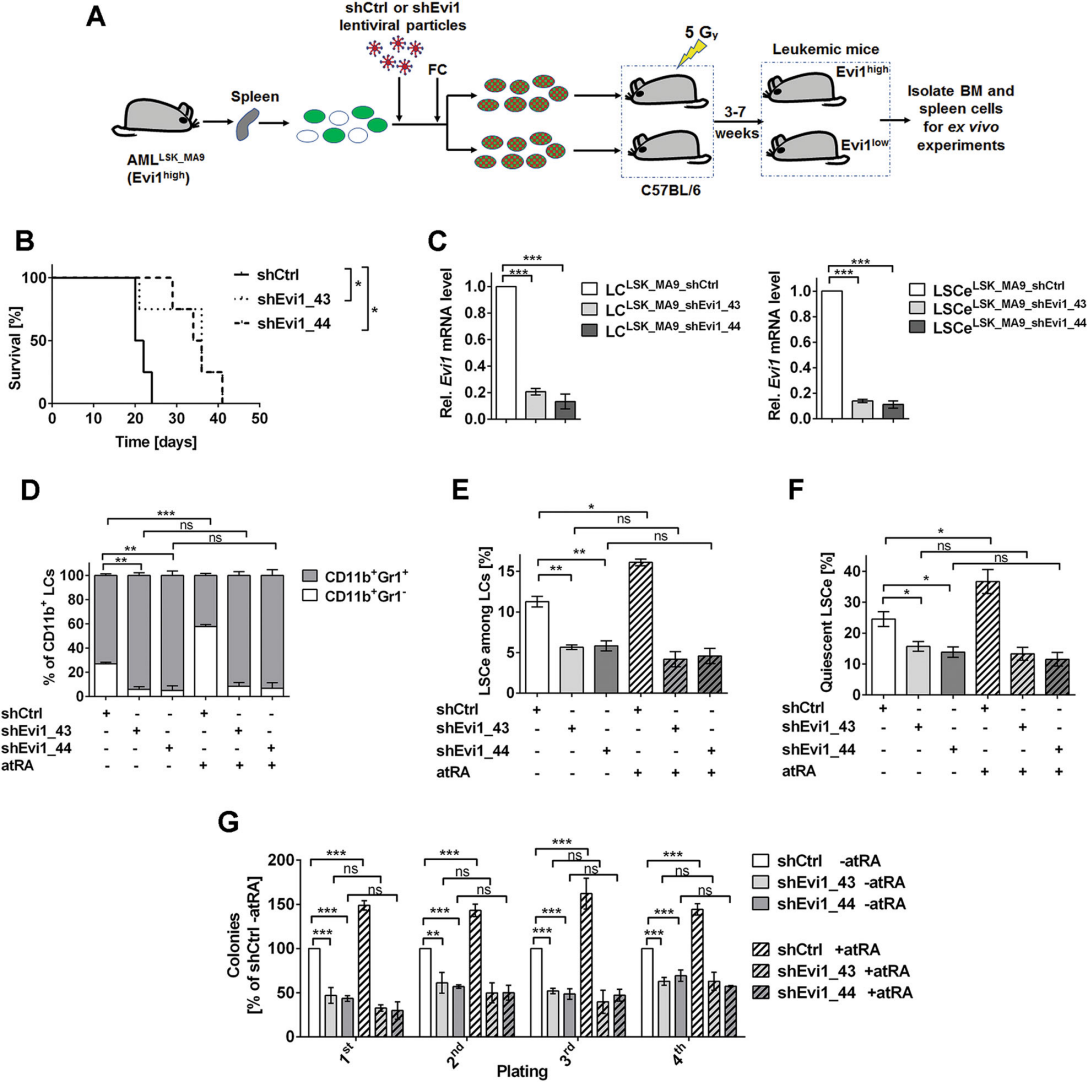
*Evi1* prevents LC maturation and promotes LSC properties, and its effects are enhanced by atRA. **a** Schematic of experimental design. FC, flow cytometry. **b** Kaplan–Meier plot of mice transplanted with shCtrl or shEvi1-transduced LC^LSK_MA9^ (250,000 cells/mouse). *n* = 4/group; *p < 0.05; log-rank test. **c** Relative *Evi1* mRNA levels in BM LC^LSK_MA9_shCtrl^ and LC^LSK_MA9_shEvi1^ (left panel) and the corresponding LSCe (right panel). qRT-PCR; *n* = 3; ***p < 0.001; *t*-test. **d**–**g** BM cells (**d**–**f**) or BM LCs (Venus^+^ RFP^+^ cells; panel **g**) from terminally ill mice were treated with 1 µM atRA or solvent for 3 days. *n* = 3; *p < 0.05; **p < 0.01; ***p < 0.001; ns, not significant; ANOVA followed by Bonferroni's post-hoc test. **d** Myeloid differentiation. **e** Proportions of LSCe among LCs. **f** Proportions of quiescent LSCe (LSCe in G_0_). **g** Colony formation in methyl cellulose, presented as percent of solvent-treated LC^LSK_MA9_shCtrl^ in each round of plating.

The previous experiment employed different cells of origin (LSK cells vs. CMPs) to generate Evi1^high^ and Evi1^low^ LCs, mimicking the situation in human *MA9*-driven AML. However, the resulting LCs differ from each other by the expression not only of *Evi1*, but of multiple genes^37^. To test whether the above described differences between LC^LSK_MA9^ and LC^CMP_MA9^ and the LSCe/LSCs therein were at least partially due to differences in *Evi1* expression, a knock-down approach was used. LC^LSK_MA9^ were transduced with lentiviral vectors containing shRNAs against *Evi1* (validated by immunoblotting, Supplementary Fig. S2A) or shCtrl, and transplanted into recipient mice (Fig. 2a). Consistent with earlier reports^12,13^, knock-down of *Evi1* significantly delayed leukemogenesis, and furthermore decreased WBC and the percentage of LCs in spleen of terminally ill mice (Fig. 2b; Supplementary Fig. S2B, C). qRT-PCR confirmed strongly decreased *Evi1* mRNA expression in LC^LSK_MA9_shEvi1^ *vs*. LC^LSK_MA9_shCtrl^ and the corresponding LSCe (Fig. 2c). While atRA increased the proportion of immature cells among LC^LSK_MA9_shCtrl^, down-regulation of *Evi1* strongly reduced the proportion of immature cells both in the absence and presence of atRA (Fig. 2d, Supplementary Fig. S2D). *Evi1* knock-down also decreased the proportion of LSCe among LCs, the fraction of LSCe in G_0_, and the activity of LSCs. Moreover, these parameters were augmented by atRA in LC^LSK_MA9_shCtrl^, but not LC^LSK_MA9_shEvi1^ (Fig. 2e–g; Supplementary Fig. S2E-G). Thus, experimental manipulation of *Evi1* expression reproduced the effects of the cell of origin (LSK cells vs. CMPs) on key LC/LSCe/LSC properties in *MA9*-driven murine AML, indicating that *Evi1* represents a central determinant of the characteristics of transformed HSCs. Furthermore, *Evi1* unleashed the ability of atRA to promote immaturity and stemness of AML.

### *Evi1* and atRA augment LSC activity and mutually enhance their effects in an in vivo limiting dilution assay

**Fig. 3:**
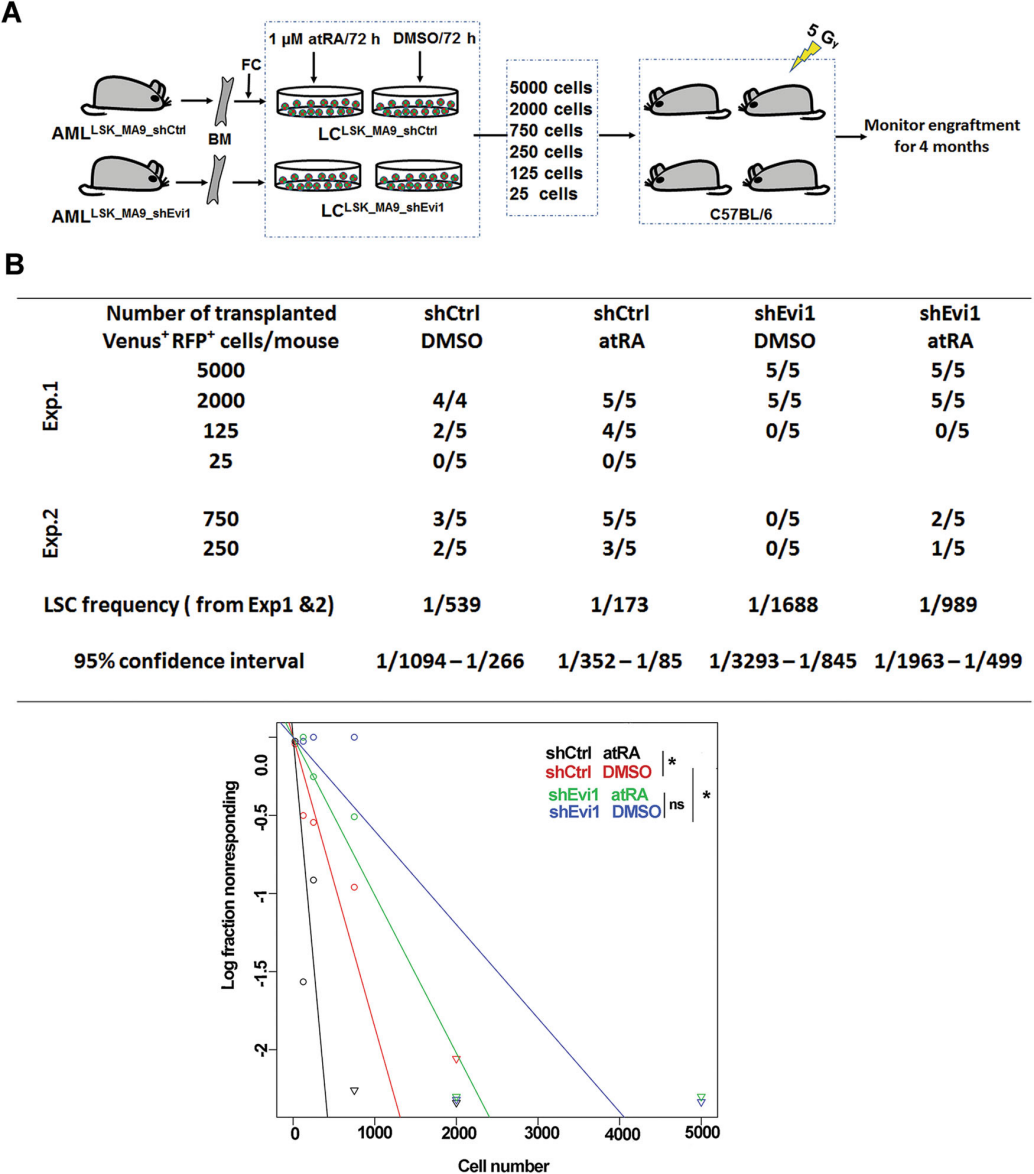
*Evi1* and atRA cooperate to augment LSC activity in an in vivo limiting dilution assay. **a** Schematic of experimental design. BM, bone marrow; FC, flow cytometry. **b** In vivo limiting dilution assay to determine the frequency of functional LSCs. Upper: table showing the numbers of responders (defined by the presence of ≥1% Venus^+^ RFP^+^ cells in BM at the time of sacrifice) and the total numbers of evaluable recipients for each cell dose. Data from two independent experiments were combined, and LSC frequencies were calculated by applying the maximum likelihood method using ELDA software. Lower: Plot showing the logarithmized fractions of non-responding recipients of different numbers of LCs. Statistical significance was assessed using the Chi-square test.

The in vivo limiting dilution assay represents the gold standard for measuring LSC activity, and was used to further confirm the effects of *Evi1* and atRA on LSCs (Fig. 3a). Knock-down of *Evi1* significantly decreased LSC frequency compared to control (Fig. 3b). Furthermore, atRA significantly enhanced LSC frequency among LC^LSK_MA9_shCtrl^. The effect of atRA in LC^LSK_MA9_shEvi1^ was substantially smaller and not significant (Fig. 3b). Taken together, these data demonstrate that *Evi1* is a critical positive regulator of LSC function, and its expression augments atRA promoted stemness in *MA9*-driven murine AML.

### EVI1 regulates a large number of genes, and enhances atRA dependent gene regulation, in LSCe

**Fig. 4:**
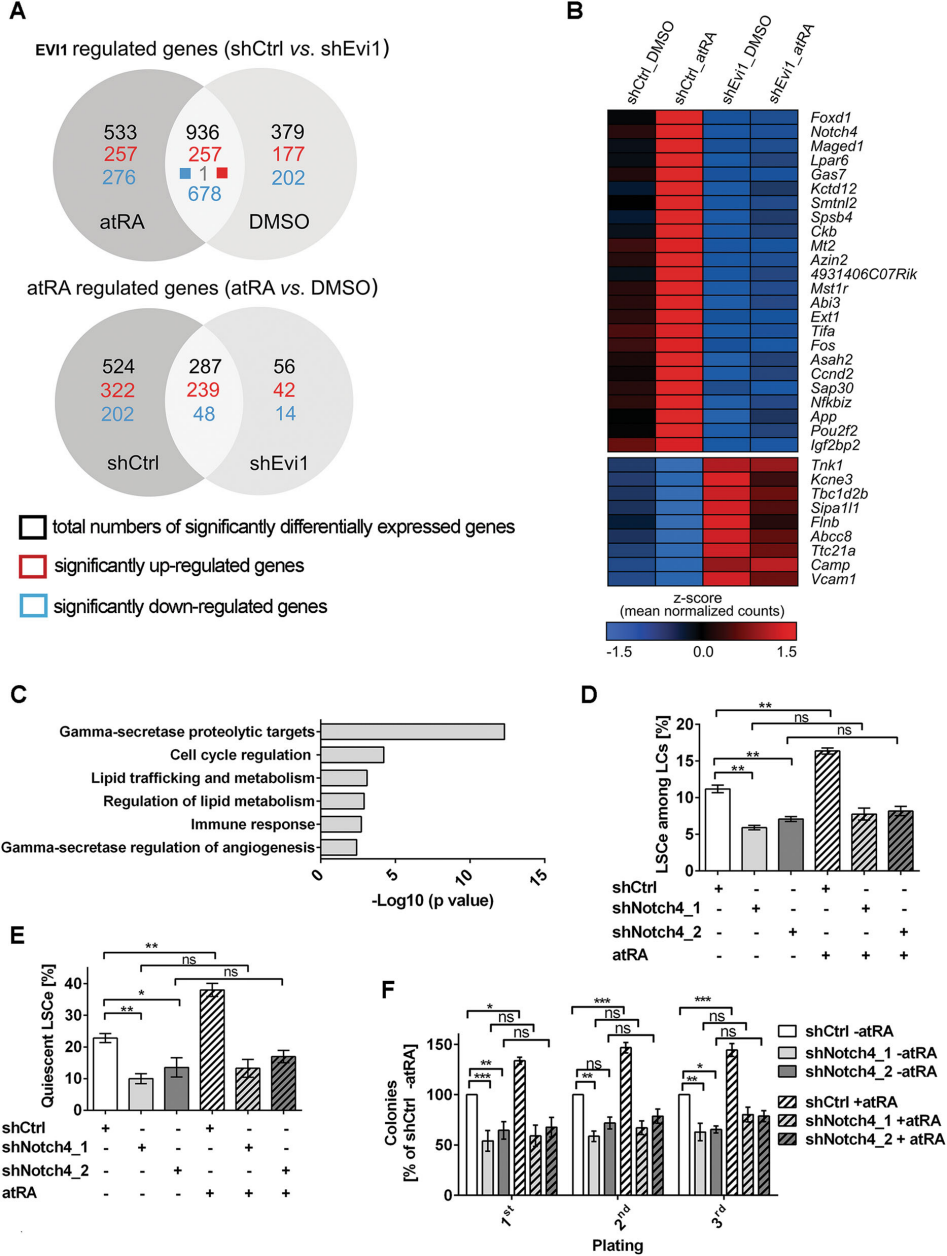
EVI1 augments transcriptional responses to atRA in LSCe, and *Notch4* is a relevant joint target. LSCe^LSK_MA9_shCtrl^ and LSCe^LSK_MA9_shEvi1^ were treated with atRA or solvent for 24 h and subjected to RNA-sequencing. **a** Numbers of genes regulated by EVI1 or atRA at an FDR < 0.05. **b** Heatmap of genes that were minimally affected by atRA in LSCe^LSK_MA9_shEvi1^, but whose regulation by EVI1 was enhanced by atRA in LSCe^LSK_MA9_shCtrl^. **c** Pathway enrichment analysis of genes shown in **b**. **d**–**f** BM cells from mice terminally ill after transplantation with *MA9* transduced LSK cells were further transduced with shCtrl, shNotch4_1, or shNotch4_2. Venus^+^ RFP^+^ cells were treated with 1 µM atRA or an equivalent amount of solvent for 3 days. *n* = 3; *p < 0.05; **p < 0.01; ***p < 0.001; ns, not significant; ANOVA followed by Bonferroni's post-hoc test. **d** Proportions of LSCe among LCs. **e** Proportions of quiescent LSCe (LSCe in G_0_). **f** Colony formation in methyl cellulose, presented as percent of solvent-treated, shCtrl transduced LC^LSK_MA9^ in each round of plating.

Both EVI1 and atRA exert their biological effects mainly through the regulation of gene transcription^9,10^. To explore the molecular basis of their impact on LSCs, RNA-seq was performed on atRA or solvent treated LSCe^LSK_MA9_shCtrl^ and LSCe^LSK_MA9_shEvi1^. 1315 and 1469 genes were significantly (FDR < 0.05) regulated by EVI1 in the absence and presence of atRA, respectively; 936 of these (71 and 64%, respectively) responded to EVI1 in either condition (Fig. 4a, Supplementary Table S2A, B). Biological pathways enriched among these genes are listed in Supplementary Table S2C. Reaffirmingly, a transcription factor analysis (Metacore) showed that the lists of genes regulated by EVI1 in LSCe were enriched for previously reported targets of EVI1 (MECOM), as well as for targets of transcription factors known to be regulated by, or to functionally interact with, EVI1 (e.g., GATA-2, c-Fos, c-Jun, RUNX1, SMAD3, and PU.1;^9,15,39–41^ Supplementary Table S2D, E). Furthermore, gene set enrichment analysis (GSEA)^42^ revealed that gene expression profiles associated with stemness and/or poor outcome in AML, as well as EVI1-dependent profiles previously identified in other experimental systems, were enriched in our EVI1-dependent signature (albeit not always in the expected direction; Supplementary Table S2F).

atRA altered the expression of 811 genes in LSCe^LSK_MA9_shCtrl^, but of only 343 genes in LSCe^LSK_MA9_shEvi1^; 287 of these genes (35 and 84%, respectively) responded in both cell types (Fig. 4a, Supplementary Table S3A, B). These numbers indicated that gene regulation by atRA in LSCe was strongly dependent on EVI1, because EVI1 down-regulation reduced the number of genes affected by atRA by more than half. Pathways enriched among atRA regulated genes are listed in Supplementary Table S3C, and GSEA results in Supplementary Table S3F. The transcription factor analysis further illustrated the strong impact of EVI1 on gene regulation by atRA: the list of genes regulated by atRA in LSCe^LSK_MA9_shCtrl^, but not the corresponding list from LSCe^LSK_MA9_shEvi1^, was enriched for reported targets of PU.1, several C/EBP family members, c-Fos, SMAD3, and GATA-2. Remarkably, even RARα, RARβ, and RXRγ targets were enriched only among genes regulated by atRA in the presence, but not the absence, of EVI1 (Supplementary Table S3D, E).

Finally, we searched for genes with a transcriptional pattern reflecting the biological response pattern to EVI1 and atRA. Genes that were only minimally affected by atRA in LSCe^LSK_MA9_shEvi1^, but that were regulated by EVI1 in a manner that was enhanced by atRA, were defined as described in the Supplementary Methods. 33 genes conformed to this definition; 24 of these were induced and 9 repressed by EVI1 (Fig. 4b; Supplementary Table S4A). Despite this small number of genes, several pathways were significantly enriched among them (Fig. 4c; Supplementary Table S4B).

Collectively, these data show that *Evi1* and atRA interacted not only to regulate key biological properties of LSCe/LSCs, but also with respect to the regulation of gene transcription.

### *Notch4* is a downstream mediator of the effects of *Evi1* and atRA on leukemic stemness

Among the genes whose up-regulation by EVI1 was enhanced by atRA, *Notch4* represented a particularly interesting candidate to mediate at least some of their biological effects on LSCs: firstly, *Notch4* responded strongly to both EVI1 and atRA (Fig. 4b; Supplementary Table S4A), secondly, *NOTCH4* expression was higher in AML compared to normal BM and to normal hematopoietic stem and progenitor cells (Supplementary Fig. S3A–C), thirdly, *NOTCH4* has previously been implicated in tumor aggressiveness^43–47^ (see Discussion for details), and fourth, *Evi1* acted through *Notch* to promote HSC emergence during zebrafish embryogenesis^48^. Joint regulation of *Notch4* by EVI1 and atRA was confirmed by qRT-PCR (Supplementary Fig. S3D). The γ-secretase inhibitor DAPT reduced LSCe/LSC abundance, quiescence, and activity in an *Evi1*-dependent manner, and diminished the effects of atRA on leukemic stemness (Supplementary Fig. S3E–I). To confirm that the effects of DAPT were specific to *Notch4* rather than other γ-secretase targets, bone marrow cells from terminally ill LSK_MA9 recipient mice were transduced with two different shRNAs against *Notch4* (shNotch4_1, shNotch4_2, validated by qRT-PCR and flow cytometry, Supplementary Fig. S3J, K) or with shCtrl. Knock-down of *Notch4* reduced the abundance, quiescence, and activity of LSCe/LSCs, and counteracted the effects of atRA on these parameters (Fig. 4d–f). Together, these data establish *Notch4* as a relevant downstream mediator of the effects of *Evi1* and atRA on LSCs.

### A pan-RAR antagonist decreases LSCe abundance and quiescence in an *Evi1*-dependent manner

**Fig. 5:**
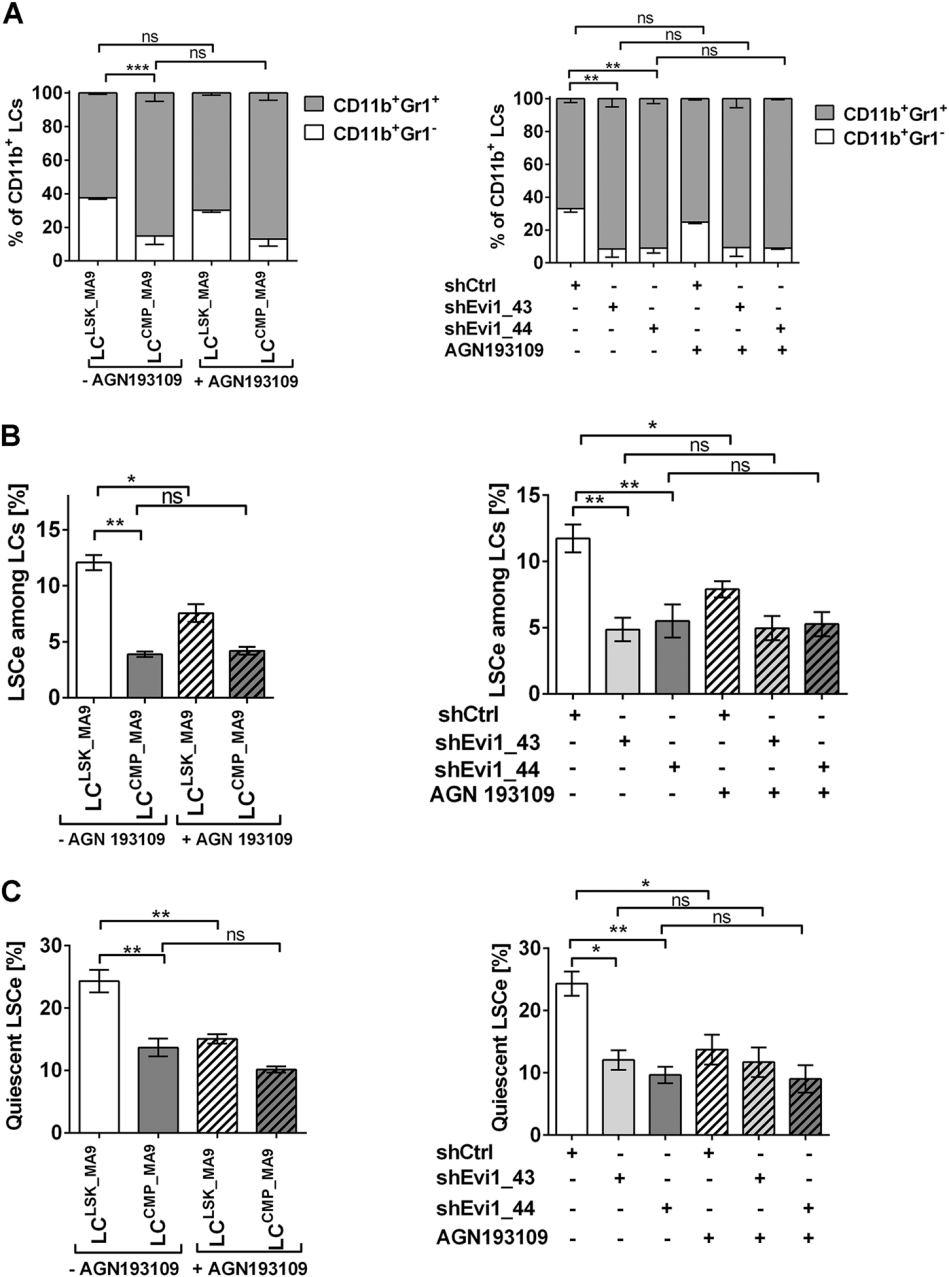
The pan-RAR antagonist AGN193109 decreases LSCe abundance and quiescence in an *Evi1-*dependent manner. BM cells from terminally ill recipients of *MA9*-transduced LSK cells or CMPs, or of shRNA transduced LC^LSK_MA9^ were treated with 1 µM AGN193109 or solvent for 3 days. *n* = 3; *p < 0.05; **p < 0.01; ns, not significant; ANOVA followed by Bonferroni's post-hoc test. **a** Myeloid differentiation. **b** Proportions of LSCe among LCs. **c** Proportions of quiescent LSCe (LSCe in G_0_).

To determine whether the above described effects of *Evi1* on LC immaturity and leukemic stemness may have been augmented by trace amounts of atRA present in the cell culture media^49^, LC^LSK_MA9^, LC^CMP_MA9^, LC^LSK_MA9_shCtrl^, and LC^LSK_MA9_shEvi1^ were treated with the pan-RAR antagonist AGN193109 or solvent for three days. AGN193109 had no significant effect on LC maturity (Fig. 5a). Regarding LSCe properties, AGN193109 decreased the abundance and quiescence of LSCe^LSK_MA9^ and LSCe^LSK_MA9_shCtrl^, but not of LSCe^CMP_MA9^ or LSCe^LSK_MA9_shEvi1^ (Fig. 5b, c). This demonstrates that the impact of atRA on LSCe was not only dependent on *Evi1*, but conversely, the effects of *Evi1* were partially dependent on activated retinoic acid receptors.

### In vivo treatment with pan-RAR antagonist delays leukemogenesis and reduces stemness of Evi1^high^ AML

**Fig. 6:**
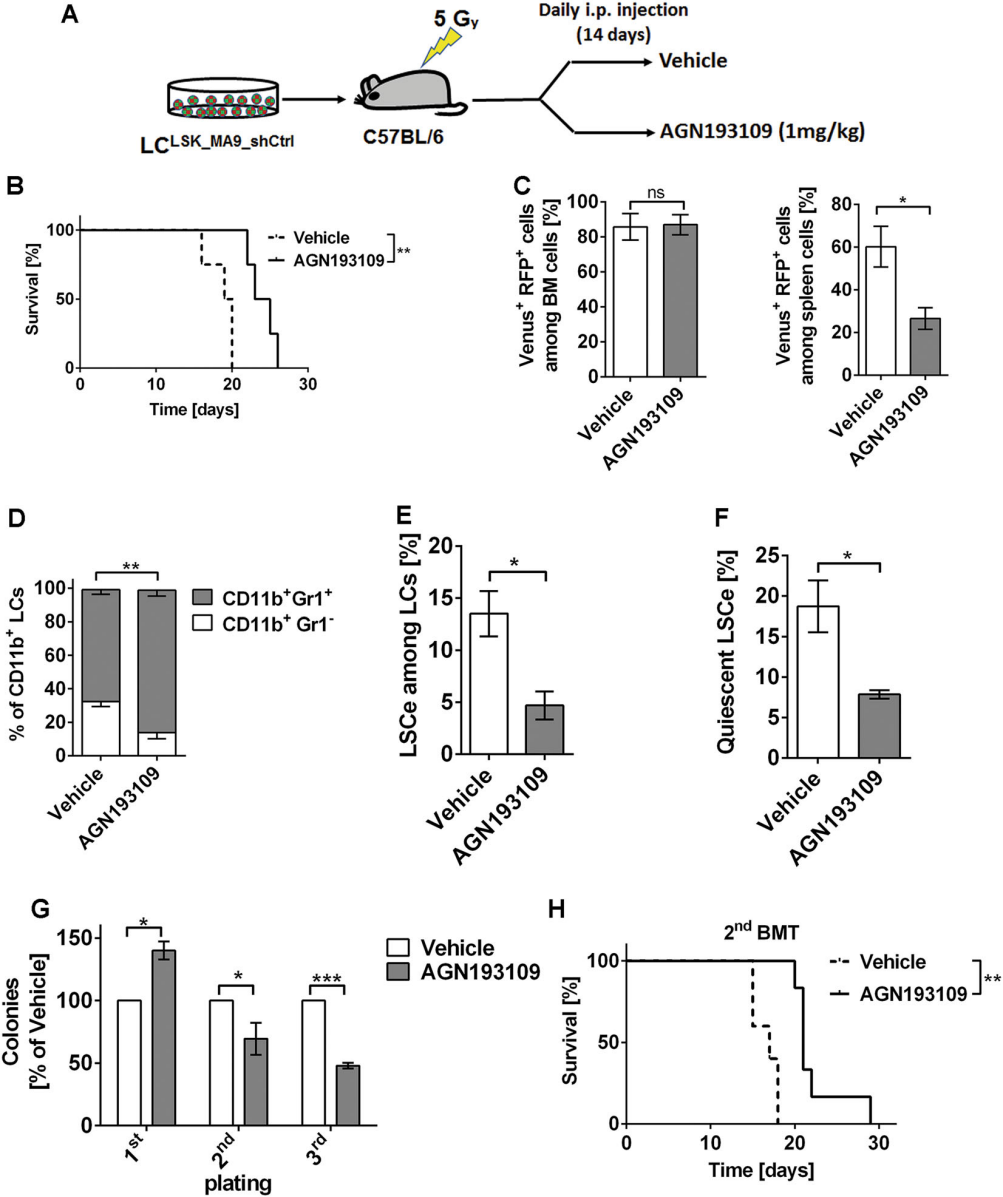
The pan-RAR antagonist AGN193109 delays leukemogenesis and decreases stemness in *MA9*-driven, Evi1^high^ murine AML. **a** Schematic of experimental design. **b** Kaplan–Meier plot of mice transplanted with LC^LSK_MA9_shCtrl^ (40,000 cells/mouse) and treated with AGN193109 (1 mg/kg) or vehicle (2.55% DMSO in PBS) by daily intraperitoneal (i.p.) injection for 14 days. *n* = 4/group; **p < 0.01; log-rank test. **c**–**f** Flow cytometric analysis of spleen (**c**) and BM cells (**c**–**f**) derived from terminally ill, AGN193109 or vehicle treated mice. LCs were defined as Venus^+^ RFP^+^ cells, but similar results were obtained if the analyses were not restricted to shCtrl expressing cells, but included all leukemic (Venus^+^) cells. *n* = 4; *p < 0.05; **p < 0.01; ns, not significant; *t*-test. **c** Percentages of LCs among BM and spleen cells. **d** Myeloid differentiation. **e** Proportions of LSCe among LCs. **f** Proportions of quiescent LSCe (LSCe in G_0_). **g** Colony formation in methyl cellulose by LCs, presented as percent of colonies from vehicle-treated mice in each round of plating. *n* = 3; *p < 0.05; ***p < 0.001; ANOVA followed by Bonferroni's post-hoc test. **h** Kaplan–Meier plot of mice transplanted with BM LCs derived from terminally ill, AGN193109 or vehicle treated mice (20,000 cells/mouse). *n* = 5/group; **p < 0.01; log-rank test. 2^nd^ BMT, secondary bone marrow transplantation.

Since atRA enhanced, and the pan-RAR antagonist AGN193109 decreased, key LSCe/LSC properties in an *Evi1* dependent manner ex vivo, we next asked whether AGN193109 would inhibit leukemia formation and stemness in vivo. Mice were transplanted with LC^LSK_MA9_shCtrl^ and treated with AGN193109 or vehicle for two weeks (Fig. 6a). Notably, AGN193109 significantly improved overall survival compared to control (Fig. 6b). Even though it had no effects on the proportion of LCs in BM, WBC, or spleen weight of terminally ill mice (Fig. 6c; Supplementary Fig. S4A, B), it decreased the percentage of LCs in spleen (Fig. 6c; Supplementary Fig. S4C) and promoted myeloid differentiation (Fig. 6d; Supplementary Fig. S4D). Furthermore, in vivo exposure to AGN193109 decreased LSCe abundance and quiescence (Fig. 6e, f; Supplementary Fig. S4E, F), and caused down-regulation of *Notch4* (Supplementary Fig. S4G). In a serial replating assay with in vivo treated cells, AGN193109 increased progenitor activity (first plating), but decreased LSC activity (replatings; Fig. 6g). Furthermore, re-transplantation experiments showed that AGN193109 treatment of primary recipients delayed leukemogenesis (Fig. 6h) and decreased spleen weight (Supplementary Fig. S4H) in secondary recipients. Taken together, in vivo treatment with AGN193109 delayed the emergence of Evi1^high^ AML, and this was associated with enhanced myeloid differentiation and decreased stemness.

### *EVI1* promotes quiescence in human AML cell lines with stem cell characteristics, and atRA enhances its effect

**Fig. 7:**
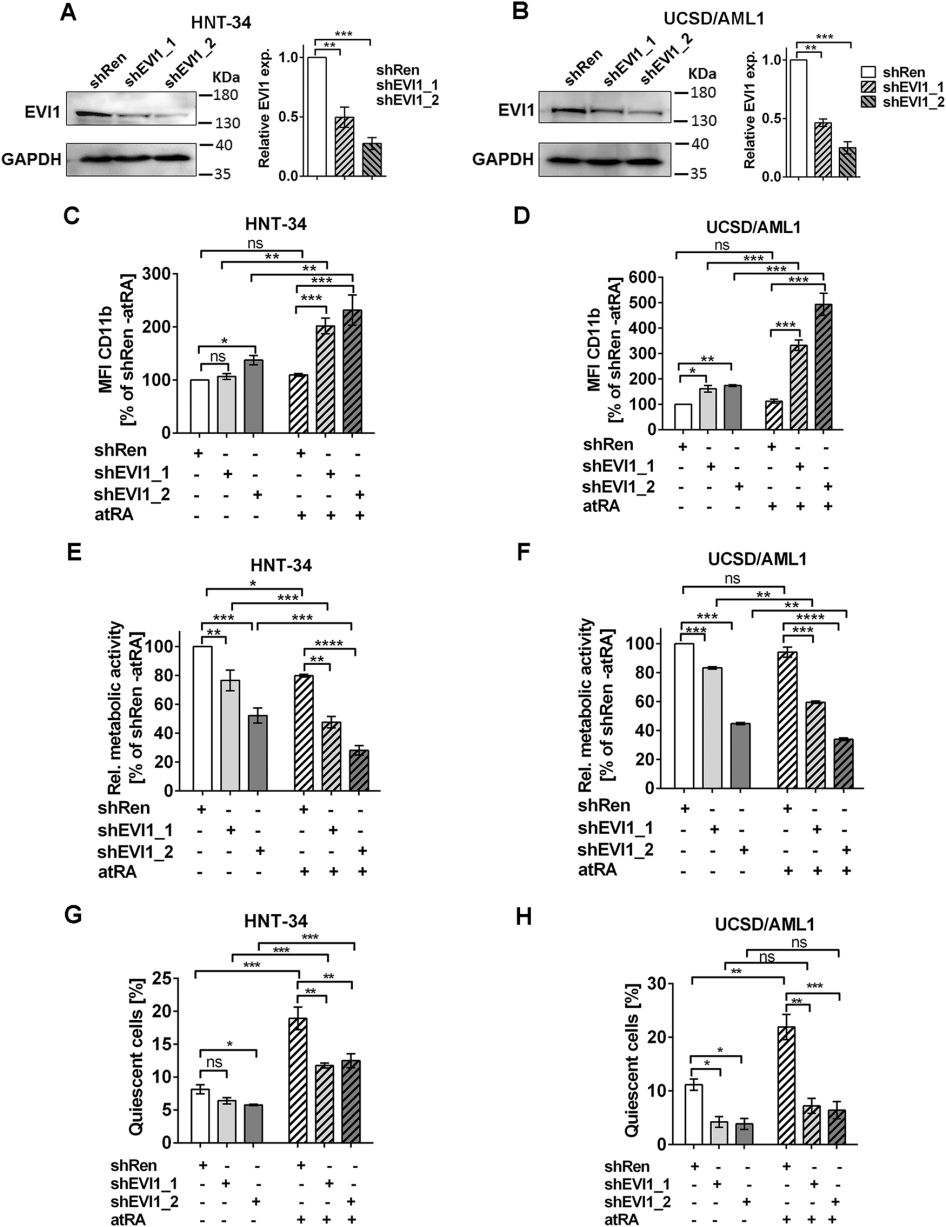
*EVI1* and atRA cooperatively promote quiescence in human myeloid cell lines with stem cell characteristics. **a**, **b** Immunoblot analyses of HNT-34 (**a**) and UCSD/AML1 (**b**) derivative cell lines. Cells were treated with doxycycline for 2 days to induce shRNAs. Left panels, representative experiments. Right panels, quantification; *n* = 3; **p < 0.01; ***p < 0.001; *t*-test. (**c**–**h**) HNT-34 and UCSD/AML1 derivative cell lines were treated with doxycycline for 2 days; 1 µM atRA or solvent was added for another 3 days. *n* = 3; *p < 0.05; **p < 0.01; ***p < 0.001; ns, not significant; ANOVA followed by Bonferroni's post-hoc test. **c**, **d** Myeloid differentiation (mean fluorescence intensity (MFI) of CD11b). HNT-34 (**c**), UCSD/AML1 (**d**). **e**, **f** Relative viability (measured using metabolic activity as a proxy). HNT-34 (**e**), UCSD/AML1 (**f**). **g**, **h** Proportion of quiescent cells (cells in G_0_). HNT-34 (**g**), UCSD/AML1 (**h**). For all panels, control experiments performed in the absence of doxycycline are shown in Supplementary Fig. S5.

To determine whether the results obtained with the *MA9* mouse model could be confirmed in human cells, the AML derived cell lines HNT-34 and UCSD/AML1 were used. Both cell lines express high levels of the stem cell gene *EVI1*, and are positive for the stem and progenitor cell associated surface marker CD34. They were transduced with lentiviral vectors expressing shRNAs targeting *EVI1* or a control shRNA (shRen) in a doxycycline-inducible manner. Immunoblot analysis confirmed the down-regulation of EVI1 in HNT-34_shEVI1 and UCSD/AML1_shEVI1 cells (Fig. 7a, b; Supplementary Fig. S5A, B). Knock-down of *EVI1* reduced CD34 expression in UCSD/AML1, but not HNT-34 cells (Supplementary Fig. S5C, D), probably reflecting differences between the two cell lines regarding the contributions of other regulators to the expression of CD34. In both cell lines, *EVI1* knock-down slightly increased mean fluorescence intensity of the myeloid differentiation marker CD11b, and re-sensitized cells to atRA-induced differentiation (Fig. 7c, d; Supplementary Fig. S5E, F). Accordingly, it decreased, and enhanced the atRA-induced reduction, in viability (measured via the proxy, metabolic activity; Fig. 7e, f; Supplementary Fig. S5G, H). Thus, like in primary murine cells, *EVI1* acted to prevent myeloid maturation in these cell lines. In contrast, atRA revealed its previously described contradictory effects^21–24^ in the two systems, preventing and promoting differentiation in primary mouse cells *vs*. human cell lines, respectively. Similar to the mouse model, *EVI1* and atRA interacted to promote quiescence of HNT-34 and UCSD/AML1 cells: down-regulation of *EVI1* decreased the proportion of cells in G_0_, and reduced the atRA-induced increase in cell quiescence (Fig. 7g, h; Supplementary Fig. S5I, J). In summary, like in primary mouse cells, the combined effects of *EVI1* and atRA in these human AML cell lines, which retain some stem cell characteristics, were the prevention of differentiation and the promotion of quiescence.

### atRA increases, and AGN193109 decreases, stemness in primary human AML samples in a manner related to the expression of *EVI1*

**Fig. 8:**
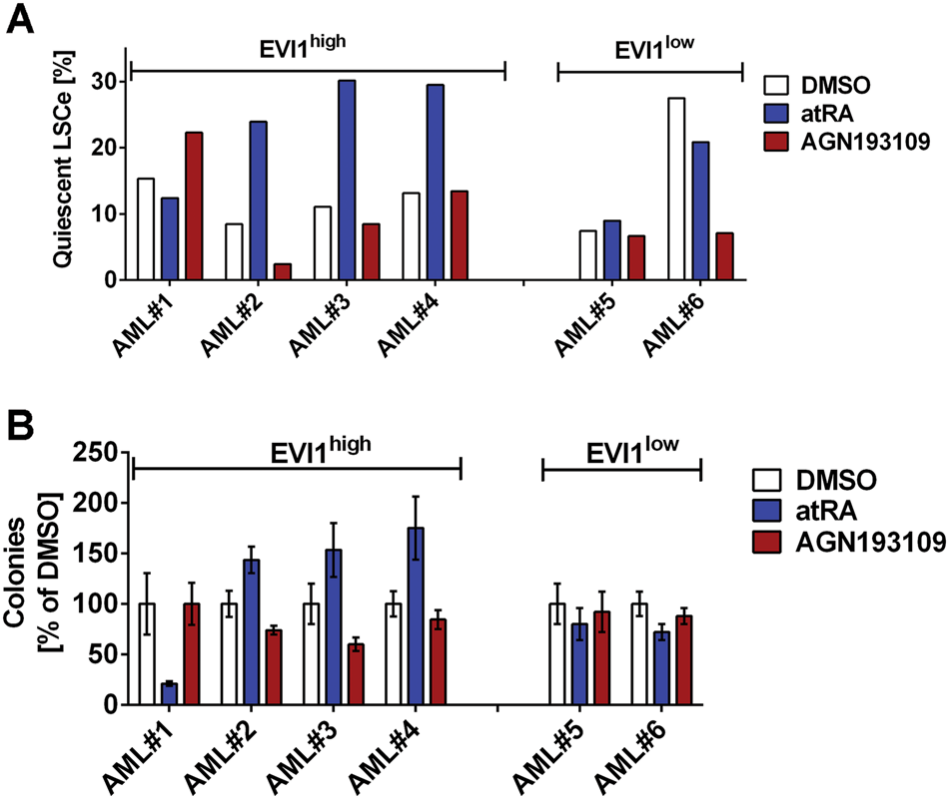
Effects of retinoids on stem cell/progenitor properties of primary AML samples with variable *EVI1* expression. Primary AML samples were treated with 1 µM atRA, 1 µM AGN193109, or corresponding amounts of solvent (DMSO) for 3 days and subjected to flow cytometric analysis or transferred to methyl cellulose. **a** Proportion of quiescent LSCe (LSCe (CD34^+^ CD38^-^ cells) in G_0_). **b** Colony formation in methyl cellulose. Mean +/- SD from duplicate measurements, presented as percent of DMSO-treated cells.

Finally, we tested the effects of atRA and AGN193109 on cell differentiation, LSCe quiescence, and stem/progenitor activity of primary human AML samples. Samples from six AML patients (four EVI1^high^ and two EVI1^low^ as determined by qRT-PCR, Supplementary Fig. S6A) were included in the study; their clinical characteristics are summarized in Supplementary Table S5. Effects of a three day treatment with atRA or AGN193109 on CD11b expression of these primary cells were small and did not show a clearly EVI1 related pattern (Supplementary Fig. S6B). In contrast, atRA enhanced the fraction of LSC enriched cells (CD34^+^ CD38^-^ cells, LSCe) in G_0_ in 3/4 EVI1^high^ samples, but had the opposite or no effect in the two EVI1^low^ samples (Fig. 8a, Supplementary Fig. S6C). Antagonist treatment did not yield a clear pattern in this assay. However, atRA increased, and AGN193109 decreased, clonogenic capacity in 3/4 EVI1^high^ samples, while in EVI1^low^ samples, atRA slightly decreased colony formation, and AGN193109 had only minimal effects (Fig. 8b). In summary, even though AGN193109 effects in these experiments were relatively small, probably reflecting low atRA concentrations in the media, our data show that atRA promotes, and the pan-RAR antagonist counteracts, stem/progenitor properties in a manner related to the expression of *EVI1* also in primary AML samples.

## Discussion

*EVI1* overexpression is associated with a particularly poor prognosis in AML^6–8^. Although its roles in leukemogenesis, normal HSCs, and chronic myeloid leukemia LSCs have been studied extensively^11–15,50^, its impact on AML LSCs, which are the key drivers of this disease^1^, has not been investigated so far. Here, we establish a previously unreported role of *Evi1* in augmenting key LSC properties in an *MA9*-driven mouse model of AML, and show that atRA further promoted AML stemness in an *EVI1*-dependent manner. Indeed, in the *MA9* model, the activity of atRA was abolished in Evi1^low^ LSC/LSCe^CMP_MA9^ or LSC/LSCe^LSK_MA9_shEvi1^. In the human AML cell lines, analogous effects of, and interactions between, *EVI1* and atRA were observed, but these were only reduced by the *EVI1* knock-down, likely due to its incompleteness. Results were further confirmed in primary AML samples with high and low *EVI1* expression, with the exception of AML #1, which expressed high levels of *EVI1*, but did not respond to atRA in the expected manner. Since AML is a genetically and molecularly heterogeneous disease, this may be due to the presence of additional lesions that may be able to counteract the effects of *EVI1* (see below).

In addition to their roles in LSCs, *EVI1* and atRA interacted with respect to LC differentiation: in the mouse model, they cooperated to maintain cells in a more immature state; in the human cell lines, *EVI1* prevented atRA-induced differentiation. Thus, in both models, atRA-treated cells that expressed *EVI1* were less mature than those not expressing this gene. No strong effect of atRA on myeloid differentiation was observed in primary AML samples. This is in contrast to a previous report^30^, which may be due to differences in incubation time, atRA dose, and/or additional genetic and molecular lesions present in the primary samples.

Several EVI1/MECOM mRNA and protein variants have been described^51^. The most abundant and best characterized of these are (i) the originally described 1051 amino acid variant usually referred to as EVI1, (ii) splice variant EVI1Δ324, which lacks part of the first of two zinc finger domains present in EVI1, and (iii) alternative promoter variant MDS1/EVI1, which has an N-terminal extension containing a PR-domain as compared to EVI1^51^. EVI1Δ324 is often co-expressed with EVI1, but appears to be of subordinate functional importance^52,53^. This is confirmed by the fact that it is not targeted by shEVI1_2, which nevertheless caused the same phenotypes as shEVI1_1 in our experiments. All other shRNAs used in this study target all three EVI1 mRNA variants. Both EVI1 and MDS1/EVI1 are expressed in the *MA9* model, but, as is usually the case in cells with 3q26 rearrangements^17,18^, only EVI1 is expressed in UCSD/AML1 and HNT-34 cells. This may indicate that the PR-domain lacking variant EVI1 causes the increased stemness and decreased differentiation observed in our model systems. This would also be in line with a widespread assumption that EVI1 is the oncogenic protein variant, while MDS1/EVI1 may act as its antagonist^54,55^. However, roles for *Mds1/Evi1* in HSCs and in *MA9*-driven murine AML have also been reported^56,57^. Therefore, future experiments will have to resolve the question which EVI1 protein variant(s) mediate(s) the phenotypes described in the present report.

The situation is even more complex for the retinoic acid receptor, which is composed of an RAR and an RXR subunit, each of which is encoded by three paralogous genes that additionally are subject to alternative splicing^58,59^. In normal hematopoiesis and an *AML1-ETO* driven mouse model of AML, atRA promoted the maintenance of stem cells and the differentiation of more mature myeloid cells through the actions of *Rarg* and *Rara*, respectively^23,38^. In an acute promyelocytic leukemia cell line, the splice variants RARA1 and RARA2 had differentiation promoting and inhibiting functions, respectively^59^. By analogy, *RARG* may mediate the effects of atRA on stemness, and *RARA* those on differentiation, in our models. However, further experiments are required to directly address this issue.

RNA-seq on atRA or solvent treated LSCe^LSK_MA9_shCtrl^ and LSCe^LSK_MA9_shEvi1^ revealed a pivotal role of EVI1 also in augmenting transcriptional effects of atRA in LSCe. Among the genes that were jointly regulated by EVI1 and atRA, *Notch4* appeared as a particularly promising candidate to mediate their biological effects in LSCs. NOTCH4 belongs to a family of transmembrane receptor proteins. *NOTCH4* up-regulation correlated to metastasis formation in melanoma^60^ and colorectal cancer^44^, and its experimental manipulation in cancer cell lines revealed a role in promoting metastasis-related properties^60–62^. *NOTCH4* was also up-regulated in high-risk B-ALL patients, and its inhibition sensitized B-ALL cells to chemotherapeutic drugs both in vitro and in vivo^47^. Experimental expression of *Notch4* inhibited myeloid differentiation and promoted HSC expansion in mice^63^. Furthermore, genetic or pharmacologic inhibition of NOTCH4 synergized with FLT3 inhibition to more effectively eliminate *FLT3*-ITD^+^ AML cells^46^. Our data extend these previous findings and suggest an important role of *Notch4* in promoting AML stemness.

Although a number of clinical trials have investigated the therapeutic potential of atRA in non-APL AML^26–28,64^, only few reports have addressed the impact of atRA on AML LSCs. atRA decreased LSC frequency in *Nup98-HoxD13*/*FLT3*-ITD-driven murine AML^65^, but had the opposite effect in murine BM cells expressing the AML1-ETO fusion protein^38^, suggesting that the effects of atRA on LSCs are strongly influenced by the identity of the respective genetic driver lesions. Supporting this notion, our study revealed that atRA promoted leukemic stemness in the *MA9* mouse model, in human AML cell lines, and in primary AML samples in an *EVI1* dependent manner. These data establish *EVI1*, a gene whose expression reflects the immaturity of the originally transformed cell at least in *MLL*-rearranged AML, as an important determinant of LSC responses to atRA.

Previously, *EVI1* and atRA were found to cooperate to enhance anti-leukemic activities in AML samples and cell lines^29,30^, while our study, focusing on LSCs, indicated the opposite, resulting in diverging assumptions about the possible utility of retinoids in the therapy of EVI1^high^ AML. In fact, patients with EVI1^high^ AML were not reported to specifically benefit from atRA in any of the pertinent clinical trials^26–28,64^. Our observation that in vivo treatment with the pan-RAR antagonist AGN193109 delayed leukemogenesis and reduced stemness in an Evi1^high^, *MA9*-driven AML model even raises the possibility that some subgroups of AML may benefit from RAR antagonists. Albeit corresponding effects of AGN193109 in primary AML cells were small, this may be due to low concentrations of atRA in the in vitro setting precluding strong antagonist effects. This assumption is supported by the finding that in the mouse model, AGN193109 effects were also more pronounced in vivo than ex vivo (Figs. 5 and 6). RAR antagonists are being explored as treatments for diverse ailments, including malignancies and hematopoietic diseases^66,67^, and did not cause any serious toxicities in corresponding mouse models^66–68^. Future studies will have to identify the most suitable antagonist (possibly RAR isoform specific^23^) for the treatment of AML. Furthermore, the extent of the therapeutic window given the role of atRA in normal HSCs^23,24^, the identity of additional AML subgroups potentially benefitting from such a therapy, and the timing of retinoid application in the context of combination therapy will have to be addressed.

In summary, we demonstrate that *EVI1* promoted key LSC properties in AML. Furthermore, atRA and *EVI1* cooperated to enhance AML stemness and to regulate gene transcription in LSCe. Their biological effects on LSCs were at least partly mediated by *Notch4*. Conversely, a pan-RAR antagonist delayed leukemogenesis and reduced stemness of EVI1^high^ AML, suggesting potential novel treatment options for this aggressive AML subtype.

## Supplementary information

Supplementary methods

Supplementary figure legends

Supplemental Table S1

Supplemental Table S5

Supplemental Figure S1

Supplemental Figure S2

Supplemental Figure S3

Supplemental Figure S4

Supplemental Figure S5

Supplemental Figure S6

Supplemental Table S2

Supplemental Table S3

Supplemental Table S4
