# Supplementary material for: All-trans retinoic acid enhances, and a pan-RAR antagonist counteracts, the stem cell promoting activity of EVI1 in acute myeloid leukemia
Source: Cell Death Dis. 2019 Dec 10;10(12):944. doi: 10.1038/s41419-019-2172-2 (PMC6904467; doi:10.1038/s41419-019-2172-2)
Supplement: Supplementary file 1 — Supplementary methods [file 41419_2019_2172_MOESM1_ESM.docx]

Supplementary method for manuscript:

***all-trans* retinoic acid enhances, and a pan-RAR antagonist counteracts, the stem cell promoting activity of *EVI1* in acute myeloid leukemia**

**Cell culture and knock-down of *EVI1* in human myeloid cell lines**

All cell lines used in this study were tested regularly for mycoplasma contamination using MycoAlert mycoplasma detection kit (Lonza), and showed negative results. Phoenix-GP and Platinum-E packaging cell lines were maintained in DMEM (Invitrogen) supplemented with 10% fetal bovine serum (FBS) (Invitrogen) and 1% Penicillin/Streptomycin (Sigma-Aldrich). HNT-34 cells ^1^ and their derivatives were grown in RPMI 1640 (Invitrogen) containing 10% FBS and 1% Penicillin/Streptomycin/Glutamine (Life Technologies). UCSD/AML1 cells ^2^ and their derivatives were grown in RPMI 1640 supplemented with 20% FBS, 1% Penicillin/Streptomycin/Glutamine, and 5 ng/ml recombinant human GM-CSF (Peprotech).

To knock down *EVI1*, shRNAs shEVI1_1 (5'-ATCTAAGGCTGAACTAGCAGA-3') and shEVI1_2 (5'-GCACTACGTCTTCCTTAAATA-3') (Open Biosystems) were cloned into LT3REVIR, a lentiviral all-in-one vector allowing for doxycycline-inducible expression of its shRNA inserts ^3^. The resulting vectors, LT3REVIR_shEVI1_1 and LT3REVIR_shEVI1_2, as well as control vector LT3REVIR_shRen, containing an shRNA against the Renilla luciferase gene, were transiently transfected into Phoenix-GP cells, along with packaging plasmids psPAX2 and pMD2.G, using the calcium chloride method. Virus-containing supernatant was harvested after 48-96 h, filtered (0.45 µm pore size), and supplemented with polybrene (4 μg/ml). HNT-34 and UCSD/AML1 cells were spinoculated at 1300 rpm for 90 min with lentiviral supernatant. The process was repeated with fresh lentiviral supernatant after 24 and 48 h. Five days after the last transduction, cells were sorted for Venus positivity on an Astrios (Beckman Coulter).

**Analysis of CD34 expression, differentiation, metabolic activity, and cell cycle distribution of human myeloid cell lines**

HNT-34 and UCSD/AML1 derivative cell lines were cultured in the presence or absence of doxycycline (1 μg/ml; Sigma-Aldrich) for 48 h to induce, or not, shRNA expression, and 1 µM atRA (Sigma-Aldrich) or corresponding amounts of solvent (DMSO; Sigma-Aldrich) were added for another 72 h. CD34 expression and myeloid differentiation were assessed by flow cytometry after staining of cells with antibodies against CD34 and CD11b (Table S1), respectively. Cellular viability (determined using metabolic activity as a proxy) was measured using the Cell Titer Glo Assay (Promega) according to the manufacturer’s instructions. To determine the cell cycle distribution, cells were fixed and permeabilized in Cytofix/Cytoperm (BD Biosciences), stained with Ki-67 antibody (Table S1) and DAPI (1 µg/mL; Sigma-Aldrich) in Perm/Wash™ Buffer (BD Biosciences), washed with Perm/Wash™ Buffer, and subjected to flow cytometry. Cells were assigned to different phases of the cycle based on DNA content. The threshold for Ki-67 positivity was determined using an isotype control antibody, and Ki-67 negative cells with a 2N DNA content were considered to be in G_0_. Flow cytometric analyses were performed using an LSR Fortessa SORP (BD Biosciences) and FlowJoX software (Treestar).

**Isolation, culture, transduction, and transplantation of primary murine cells**

Bone marrow (BM) single cell suspensions were obtained by flushing femora and tibiae of 6-8 week old C57BL/6 mice (Department of Laboratory Animal Science & Genetics, Himberg, Austria) or of terminally ill recipient mice with RPMI supplemented with 10% FBS and 1% penicillin/streptomycin, and filtering the eluate through a 40 µm cell strainer (Falcon, Corning, NY, USA). Spleen cells were harvested from terminally ill recipient mice by crushing the organ with the plunger of a 5 ml syringe and filtering cell suspensions through a 40 µm cell strainer. Red blood cells (RBCs) were lysed for 2 min at room temperature in RBC lysis buffer (Sigma-Aldrich).

Primary murine hematopoietic cells were cultured in IMDM medium (Thermo Fisher Scientific) containing 10% FBS, 1% L-Glutamine (Thermo Fisher Scientific), 50 ng/ml mSCF, 10 ng/ml mIL-3, 10 ng/ml mTPO, 10 ng/ml mFlt3L (all from Peprotech), and 10 ng/ml mIL-6 (Biolegend). To generate mice with *MLL-AF9* (*MA9*) driven AML, a retroviral transduction/transplantation approach was used ^4,5^. Hematopoietic stem cell enriched Lin^-^ Sca-1^+^ c-Kit^+^ (LSK) cells and common myeloid progenitors (CMPs, Lin^-^ Sca-1^-^ cKit^+^ CD34^+^ CD16/CD32^low^ cells) were obtained from BM cells of 6-8 week old female C57BL/6 mice by flow cytometry. Retroviral particles were produced by calcium chloride mediated co-transfection of Platinum-E cells with pMSCV_MA9_IRES_Venus ^6^ and the ecotropic packaging plasmid psi2 (containing the *gag*, *pol*, and *env* genes). LSK cells and CMPs (100 000 cells in 500 µl culture medium) were spinoculated with 1500 µl retroviral supernatant in the presence of 4 µg/ml polybrene (Sigma-Aldrich) and cytokines (as in the culture medium) for 1 h at 1300 rpm and 32° C in a 12-well plate precoated with Retronectin (Takara). This process was repeated with fresh retroviral supernatant after 5, 10, and 24 h, followed by a 48 h incubation of the resulting LSK_MA9 and CMP_MA9 cells in culture medium prior to transplantation.

For knock-down of *Evi1*, three different shRNAs (shEvi1_41 (5'-TTAACTGGAAGTCCAATTTAA-3'), shEvi1_43 (5'-CTCAATCAATGTACCCATTTCCT-3'), and shEvi1_44 (5'-TTTACAGAGATCCGCAATTTC-3')), as well as non-target control shRNA SHC012 (shCtrl) in pLKO.1_puro_CMV_TagRFP (Sigma-Aldrich) were transfected into Phoenix-GP cells, along with the packaging plasmids pSPAX2 and pMDG.2, using calcium chloride precipitation. Lentiviral particles were harvested after 48-72 h and used for spinoculation of spleen cells from terminally ill LSK_MA9 recipient mice as described above, except that only two transduction cycles with an interval of 24 h were performed. 3 days later, fluorescence marker positive (Venus^+^ RFP^+^) cells were sorted, allowed to recover in culture medium for 48 h, and transplanted into recipient mice.

For transplantation, 6-8 week old female C57BL/6 recipient mice were sub-lethally irradiated (5 Gy), anaesthesized on the next day, and injected retro-orbitally with LSK_MA9 or CMP_MA9 (300 000 cells/mouse; unsorted because of the strong selective advantage associated with *MA9* expression) or with shCtrl or shEvi1-transduced LC^LSK_MA9^ (250 000 Venus^+^ RFP^+^ cells/mouse). BM and spleen cells were harvested from terminally ill mice for use in *ex vivo* experiments.

To knock down *Notch4*, bone marrow cells from terminally ill LSK_MA9 recipient mice were transduced with pLKO.1_puro_CMV_TagRFP containing shNotch4_1 (5'-TGTGAGGTGGAGGTCAATGCT-3'), shNotch4_2 (5'-CTCGGTTGTAAGAAATCTGAA-3'), or shCtrl. Venus^+^ RFP^+^ cells were sorted by flow cytometry and used for stem cell assays.

**Quantitative RT-PCR (qRT-PCR)**

Total RNA was extracted from LC^LSK_MA9^, LC^CMP_MA9^, LC^LSK_MA9_shCtrl^, LC^LSK_MA9_shEvi1^, and the corresponding LSCe using Trizol, and reverse transcribed using random hexamer primers and M-MLV reverse transcriptase (all from Life Technologies). qRT-PCR was performed on a Step One Plus Real Time PCR system (Life Technologies) using GoTaq qPCR Master Mix (Promega) and the following primers: *Evi1*_fwd: 5'-CTCGAAGCCTTCAGGAACAC-3', *Evi1*_rev: 5'-AGCTTCAAGCGGGTCAGTTA-3'; *Notch4*_fwd: 5'-CTCTGCAGCCCTGGCTATAC-3'; *Notch4*_rev: 5'-GGCATCGAGCAGTGTGTG-3'; *β-2-microglobulin*_fwd: 5'-CCTTCAGCAAGGACTGGTCT-3', *β-2-microglobulin*_rev: 5'-TGTCTCGATCCCAGTAGACG-3'*.* For primary AML samples, TaqMan Gene Expression Master Mix and TaqMan probes (*MECOM* (Hs00602795_m1), *β-2-microglobulin* (Hs99999907_m1), Applied Biosystems) were used. Assays were performed in triplicate, and *Evi1* expression was normalised to *β-2-microglobulin* expression using the ΔΔC_T_ method ^7^.

**Immunoblot analysis**

To validate the shRNAs against murine *Evi1*, HEK293T cells were transiently co-transfected with pMSCV_Flag-Evi1_IRES_GFP (kindly provided by Dr. Takuro Nakamura, Cancer Institute of JFCR, Tokyo, Japan) and shEvi1_41 (5'-TTAACTGGAAGTCCAATTTAA-3'), shEvi1_42 (5'-GCAACCTTCAGCGACACATTC-3'), shEvi1_43 (5'-CTCAATCAATGTACCCATTTCCT-3'), shEvi1_44 (5'-TTTACAGAGATCCGCAATTTC-3'), shEvi1_97 (5'-GCAAATACTGTGATAGATCAT-3'), or shCtrl in pLKO.1_puro_CMV_TagRFP. 48 h later, protein lysates were collected. Knock-down of *EVI1* in human myeloid cell lines was confirmed using protein lysates from the respective HNT-34 and UCSD/AML1 derivative lines. Preparation of protein lysates, SDS-PAGE, transfer to PVDF membranes (Hybond-P; Amersham), and incubations with antibodies (Table S1) were performed using standard procedures. Blots were developed using SuperSignal West Femto or Pico Chemiluminescent Substrates (both from Thermo Fisher Scientific) and scanned using a ChemiDoc Touch Imaging System (Bio Rad). Densitometric analysis was performed with Image-J software (National Institutes of Health).

**RNA sequencing (RNA-seq) and bioinformatics analyses**

LSCe^LSK_MA9_shCtrl^ (three different mice) and LSCe^LSK_MA9_shEvi1^ (shEvi1_41, shEvi1_43, and shEvi1_44) were isolated from spleens of terminally ill mice, recovered for 24 h, and incubated with 1 µM atRA or the corresponding amount of DMSO for another 24 h. Down-regulation of *Evi1* in LSCe^LSK_MA9_shEvi1^ was confirmed by qRT-PCR. The effects of shEvi1_41 on LC differentiation and on LSCe abundance and quiescence were comparable to those of shEvi1_43 and shEvi1_44. Total RNA was isolated using QIAshredder 50 columns (Qiagen) and the RNeasy Mini Kit 50 including DNase treatment (Qiagen), and submitted to the Biomedical Sequencing Facility at the Center for Molecular Medicine (CeMM), Vienna, Austria. The amounts of total RNA were measured using the Qubit Fluorometric Quantitation system (Life Technologies), and RNA integrity was assessed using the Experion Automated Electrophoresis System (Bio-Rad). RNA-seq libraries were prepared with the TruSeq Stranded mRNA LT sample preparation kit (Illumina) using Sciclone and Zephyr liquid handling robotics (Perkin Elmer). Library concentrations were determined with the Qubit Fluorometric Quantitation system and the size distribution was assessed using the Experion Automated Electrophoresis System. Sequencing libraries were pooled, diluted, and sequenced on an Illumina HiSeq 3000 using 50 bp single read chemistry. Base calls provided by the Illumina Real-Time Analysis software were converted into BAM format (Illumina2bam) before de-multiplexing (BamIndexDecoder) into individual, sample-specific BAM files via Illumina2bam tools (<https://github.com/wtsi-npg/illumina2bam>). BAM files were converted into FASTQ files using Picard tools (2.16.0). Adapters were removed and reads were cropped at 65 bp using Trimmomatic (0.36) ^8^. Sequencing quality was checked using FASTQC (0.11.5). Reads were mapped onto mouse genome version mm10 (UCSC) using STAR aligner (2.6.0c) ^9^, RefSeq gene annotation, and 100 bp splice junction overhangs. HTSeq ^10^ was used to quantify raw gene counts. Principal component analysis (PCA) based on the 1 000 genes with the highest variability (interquartile range) of normalized regularized-log data (DESeq2) across all samples revealed that the mean distances of the LSCe^LSK_MA9_shEvi1_43^ samples from the LSCe^LSK_MA9_shEvi1_41^ and LSCe^LSK_MA9_shEvi1_44^ samples were greater than two times the intragroup standard deviation between the latter two sample types in a plot including the first two principal components. We therefore excluded LSCe^LSK_MA9_shEvi1_43^ samples from further analyses. DESeq2 ^11^ within the R environment (3.5.1) was used to identify genes differentially expressed between the four different conditions using negative binomial distribution and filtering for genes with normalized mean counts >10 across the samples entering the respective comparison. A false discovery rate (FDR) ^12^ of <0.05 was applied to identify differentially expressed genes as follows: genes regulated by EVI1 in the absence or presence of atRA (Evi1 regulation: Er_D, shCtrl_DMSO *vs* shEvi1_DMSO; Er_A, shCtrl_atRA *vs* shEvi1_atRA), as well as genes regulated by atRA in the absence or presence of EVI1 (atRA regulation: Ar_shE, shEvi1_atRA *vs.* shEvi1_DMSO; Ar_shC, shCtrl_atRA *vs.* shCtrl_DMSO). Furthermore, using customized PERL scripts, genes were identified whose expression patterns mirrored the observed biological effects, i.e., which showed little or no regulation by atRA in shEvi1 cells, and whose regulation by EVI1 was enhanced by atRA (Er_D/Ar_shC). This was achieved by applying the following criteria: for genes whose up-regulation by EVI1 was augmented by atRA, 1) Ar_shE fold-change <1.5, 2) Er_D fold-change >1.5, p <0.05, and 3) Ar_shC fold-change >1.5, p <0.05, and conversely for genes whose down-regulation by EVI1 was augmented by atRA, 1) Ar_shE fold-change >0.66, 2) Er_D fold-change <0.66, p <0.05, and 3) Ar_shC fold-change <0.66, p <0.05. Heatmaps were generated using Genesis (1.8.1) ^13^ based on z-score transformation of mean normalized counts across all four conditions.

For pathway analyses and identification of transcription factors potentially explaining the observed gene expression patterns, MetaCore version 6.36 (Clarivate Analytics) was used. Pathway analyses on EVI1 regulated genes and on atRA regulated genes were performed by using Er_D and Er_A gene lists, or Ar_shE and Ar_shC gene lists as joint input; the Er_D/Ar_shC gene list was analyzed on its own. An FDR <0.1 was considered statistically significant. Transcription factor analysis was performed on each gene list separately. Enrichment of the Er_D and Ar_shC signatures for gene expression profiles associated with stemness and/or poor outcome in AML, and for previously identified EVI1-dependent gene expression profiles, was tested using gene set enrichment analysis (GSEA) ^14^. Genes in human-derived profiles were converted into their mouse orthologs using HomoloGene.

**References:**

1. Hamaguchi, H.*, et al.* Establishment of a novel human myeloid leukemia cell line (HNT-34) with t(3;3)(q21;q26), t(9;22)(q34;q11) and the expression of EVI1 gene, p210 and p190 BCR/ABL chimaeric transcripts from a patient with AML after MDS with 3q21q26 syndrome. *Br J Haematol* **98**, 399-407 (1997).

2. Oval, J., Smedsrud, M. & Taetle, R. Expression and regulation of the evi-1 gene in the human factor-dependent leukemia cell line, UCSD/AML1. *Leukemia* **6**, 446-451 (1992).

3. Fellmann, C.*, et al.* An optimized microRNA backbone for effective single-copy RNAi. *Cell Rep* **5**, 1704-1713 (2013).

4. Krivtsov, A.*, et al.* Cell of origin determines clinically relevant subtypes of MLL-rearranged AML. *Leukemia* **27**, 852-860 (2013).

5. Nguyen, C.H.*, et al.* SOCS2 is part of a highly prognostic 4-gene signature in AML and promotes disease aggressiveness. *Sci Rep* **9**, 9139 (2019).

6. Zuber, J.*, et al.* An integrated approach to dissecting oncogene addiction implicates a Myb-coordinated self-renewal program as essential for leukemia maintenance. *Genes Dev* **25**, 1628-1640 (2011).

7. Livak, K. & Schmittgen, T. Analysis of relative gene expression data using real-time quantitative PCR and the 2(-Delta Delta C(T)) Method. *Methods* **25**, 402-408 (2001).

8. Bolger, A., Lohse, M. & Usadel, B. Trimmomatic: a flexible trimmer for Illumina sequence data. *Bioinformatics* **30**, 2114-2120 (2014).

9. Dobin, A.*, et al.* STAR: ultrafast universal RNA-seq aligner. *Bioinformatics* **29**, 15-21 (2013).

10. Anders, S., Pyl, P. & Huber, W. HTSeq--a Python framework to work with high-throughput sequencing data. *Bioinformatics* **31**, 166-169 (2015).

11. Love, M., Huber, W. & Anders, S. Moderated estimation of fold change and dispersion for RNA-seq data with DESeq2. *Genome Biol* **15**, 550 (2014).

12. Benjamini, Y. & Hochberg, Y. Controlling the false discovery rate: a practical and powerful approach to multiple testing. *J. R. Statist. Soc. B* **57**, 289-300 (1995).

13. Sturn, A., Quackenbush, J. & Trajanoski, Z. Genesis: cluster analysis of microarray data. *Bioinformatics* **18**, 207-208 (2002).

14. Subramanian, A.*, et al.* Gene set enrichment analysis: a knowledge-based approach for interpreting genome-wide expression profiles. *Proc Natl Acad Sci U S A* **102**, 15545-15550 (2005).
