# Supplementary material for: All-trans retinoic acid enhances, and a pan-RAR antagonist counteracts, the stem cell promoting activity of EVI1 in acute myeloid leukemia
Source: Cell Death Dis. 2019 Dec 10;10(12):944. doi: 10.1038/s41419-019-2172-2 (PMC6904467; doi:10.1038/s41419-019-2172-2)
Supplement: Supplementary file 2 — Supplementary figure legends [file 41419_2019_2172_MOESM2_ESM.docx]

**Supplementary table and figure legends**

**Supplementary Table S1:** Antibodies used for immunoblot analysis and flow cytometry.

**Supplementary Table S2:** (A-B) Genes regulated by EVI1 in the absence (Er_D, data sheet A) or presence (Er_A, data sheet B) of atRA; (C) pathways enriched among these genes (Metacore); (D-E) transcription factors whose previously reported targets were enriched among these genes (Metacore); (F) enrichment of gene expression profiles associated with stemness and/or outcome in AML, and of previously identified EVI1-dependent profiles, in the Er_D signature.

**Supplementary Table S3:** (A-B) Genes regulated by atRA in the absence (Ar_shE, data sheet A) or presence (Ar_shC, data sheet B) of EVI1; (C) pathways enriched among these genes (Metacore); (D-E) transcription factors whose previously reported targets were enriched among these genes (Metacore); (F) enrichment of gene expression profiles associated with stemness and/or outcome in AML, and of previously identified EVI1-dependent profiles, in the Ar_shC signature.

**Supplementary Table S4:** (A) Genes whose expression was minimally affected by atRA in LSCe^LSK_MA9_shEvi1^, but whose regulation by EVI1 was enhanced by atRA in LSCe^LSK_MA9_shCtrl^ (Er_D/Ar_shC); (B) pathways enriched among these genes.

**Supplementary Table S5:** Clinical characteristics of *de novo* AML patients whose samples were used in this study. BM, bone marrow; PB, peripheral blood; F, female; M, male; Dx, diagnosis; FAB, French American British classification; Hb, hemoglobin; PLT, platelets; WBC, white blood cell count. *EVI1* mRNA levels were determined by qRT-PCR. All samples were from the time of diagnosis. Lack of additional material prohibited testing for recurrent AML associated mutations.

**Supplementary Figure S1: *MA9*-transduced LSK cells and CMPs cause AML-like disease differing by LSCe abundance and atRA responsiveness.** (A) Gating strategy for isolation of LSK cells and CMPs by flow cytometry; MPP, multi-potent progenitors; LSK, Lineage^-^ Sca-1^+^ c-Kit^+^; CMP, common myeloid progenitors; MEP, megakaryocyte/erythrocyte progenitors; GMP, granulocyte/macrophage progenitors. (B) Flow cytometric analysis of BM LC^LSK_MA9^ and LC^CMP_MA9^ confirmed the expression of myeloid (CD11b, Gr-1) and the absence of lymphoid (CD3, B220) markers; representative experiment. (C) White blood cell counts (WBC) in peripheral blood of mice terminally ill after transplantation with LSK_MA9 and CMP_MA9. n=3; **, p<0.01; t-test. (D) Representative flow cytometric analyses of myeloid differentiation (Gr1^+^ cells among CD11b^+^ cells) after three days of treatment with atRA or solvent. Cells were pre-gated on the Venus^+^ population. Cut-offs for marker positivity were determined using isotype controls (top panel). (E) Myeloid differentiation after seven days of treatment with atRA or solvent; n=3. (F) Representative flow cytometric analyses of LSCe (Lin^-^ c-Kit^+^ Sca-1^-^ CD34^+^ CD16/CD32^hi^ cells) among LCs after three days of treatment with atRA or solvent. Cells were pre-gated on the Venus^+^ Lin^-^ c-Kit^+^ Sca-1^-^ population. Gates were set using the respective isotype control antibodies (top panel). (G) Proportions of LSCe among BM LCs after incubation with atRA or solvent for up to six days; n=2. (E, G) *, p<0.05; **, p<0.01, ns, not significant; ANOVA followed by Bonferroni's post-hoc test. In panel G, significance indicators reflect the higher of the two p-values at the three and six day time points. (H) Representative flow cytometric analyses of cell cycle distribution of LSCe after three days of treatment with atRA or solvent. Cells were pre-gated on the Venus^+^ Lin^-^ c-Kit^+^ Sca-1^-^ CD34^+^ CD16/CD32^hi^ population. The cut-off for Ki-67 negativity was determined using an isotype control antibody (top panel).

**Supplementary Figure S2: Knock-down of *Evi1* in LC^LSK_MA9^ reduces AML aggressiveness, LSCe abundance, and LSCe responsiveness to atRA.** (A) Validation of shRNAs against *Evi1*. HEK293T cells were co-transfected with pMSCV_Flag-Evi1_IRES_GFP and control or *Evi1* shRNAs. EVI1 expression was detected by immunoblot analysis using a FLAG antibody. (B, C) White blood cell counts (WBC, panel B) in peripheral blood, and percentages of LCs (Venus^+^ RFP^+^ cells, panel C) in spleens of mice terminally ill after transplantation with shCtrl or shEvi1 transduced LC^LSK_MA9^. n=3; *, p<0.05; **, p<0.01; ANOVA followed by Bonferroni's post-hoc test. (D) Representative flow cytometric analyses of myeloid differentiation (Gr1^+^ cells among CD11b^+^ cells) after three days of treatment with atRA or solvent. Cells were pre-gated on the Venus^+^ RFP^+^ population. Cut-offs for marker positivity were determined using isotype control antibodies (top panel). (E) Representative flow cytometric analyses of LSCe (Lin^-^ c-Kit^+^ Sca-1^-^ CD34^+^ CD16/CD32^hi^ cells) among LCs after three days of treatment with atRA or solvent. Cells were pre-gated on the Venus^+^ RFP^+^ Lin^-^ c-Kit^+^ Sca-1^-^ population. Gates were set using the respective isotype control antibodies (top panel). (F) Proportions of LSCe among BM LCs treated with atRA or solvent for up to six days. n=2; *, p<0.05; **, p<0.01, ns, not significant; ANOVA followed by Bonferroni's post-hoc test. Significance indicators reflect the higher of the two p-values at the three and six day time points. (G) Representative flow cytometric analyses of cell cycle distribution of LSCe after three days of treatment with atRA or solvent. Cells were pre-gated on the Venus^+^ RFP^+^ Lin^-^ c-Kit^+^ Sca-1^-^ CD34^+^ CD16/CD32^hi^ population. The cut-off for Ki-67 negativity was determined using an isotype control antibody (top panel).

**Supplementary Figure S3: *NOTCH4* expression in human AML and in LSCe from *MA9* driven murine AML, and effects of γ-secretase inhibition on leukemic stemness.** (A, B) *NOTCH4* mRNA levels in AML and in healthy bone marrow (hBM) cells. (A) GSE13159. (B) Comparison of one normal BM dataset to 10 AML datasets (<https://hgserver1.amc.nl/cgi-bin/r2/main.cgi>). (C) *NOTCH4* mRNA levels in AML and in various normal hematopoietic cell populations. HSC, hematopoietic stem cells; MPP, multi-potent progenitors; CMP, common myeloid progenitors; MEP, megakaryocyte/erythrocyte progenitors; GMP, granulocyte/macrophage progenitors (bloodspot.binf.ku.dk). (A-C) ns, not significant; *, p<0.05; **, p<0.01; ****, p<0.0001; ANOVA followed by Bonferroni's post-hoc test. (D) Relative *Notch4* mRNA levels in LSCe^LSK_MA9_shCtrl^ and LSCe^LSK_MA9_shEvi1^ treated with atRA or solvent for 24 h. n=3; ns, not significant; ***, p<0.001; ****, p<0.0001; ANOVA followed by Bonferroni's post-hoc test. (E-G) BM cells (E, F) or BM LCs (Venus^+^ RFP^+^ cells; panel G) from terminally ill recipients of shRNA transduced LC^LSK_MA9^ were treated with 5 µM γ-secretase inhibitor (DAPT) or solvent for 3 days. n=3; *, p<0.05; **, p<0.01; ***, p<0.001; ns, not significant; ANOVA followed by Bonferroni's post-hoc test. (E) Proportions of LSCe among LCs. (F) Proportions of quiescent LSCe (LSCe in G_0_). (G) Colony formation in methyl cellulose, presented as percent of solvent-treated LC^LSK_MA9_shCtrl^ in each round of plating. (H, I) BM cells from terminally ill LSK_MA9 and CMP_MA9 recipient mice were treated for 3 days with 1 µM atRA, 5 µM DAPT, and/or solvent as indicated. n=3; **, p<0.01; ***, p<0.001; ns, not significant; ANOVA followed by Bonferroni's post-hoc test. (H) Proportions of LSCe among LCs. (I) Proportions of quiescent LSCe (LSCe in G_0_). (J, K) BM cells from terminally ill LSK_MA9 recipient mice were further transduced with shCtrl, shNotch4_1, or shNotch4_2. Venus^+^ RFP^+^ cells were sorted for downstream experiments. **, p<0.01; ***, p<0.001; ANOVA followed by Bonferroni's post-hoc test. (J) Relative *Notch4* mRNA levels (qRT-PCR); n=2. (K) Expression of NOTCH4. Left panel, representative flow cytometric analysis. Right panel, mean fluorescence intensities (MFI) of NOTCH4; n=3.

**Supplementary Figure S4: *In vivo* treatment with pan-RAR antagonist increases leukemic differentiation and decreases LC frequency, LSCe abundance, and LSCe quiescence.** C57BL/6 mice were transplanted with LC^LSK_MA9_shCtrl^, and, starting 1 week later, treated with the pan-RAR antagonist AGN193109 or vehicle for 2 weeks. (A) White blood cell counts (WBC) in peripheral blood of terminally ill, vehicle or AGN193109 treated mice. (B) Spleen weight of terminally ill, vehicle or AGN193109 treated mice. (A-B) n = 4; ns, not significant. (C-F) Flow cytometric analyses of spleen (C) and BM (D-F) cells derived from terminally ill, vehicle or AGN193109 treated mice; representative experiments. Gates were set using non-transduced spleen cells (C), or cells stained with isotype control antibodies (D-F). (C) Frequency of LCs (Venus^+^ RFP^+^ cells) among spleen cells. (D) Myeloid differentiation (Gr1^+^ cells among CD11b^+^ cells) of BM LCs. Cells were pre-gated on the Venus^+^ RFP^+^ population. (E) Abundance of LSCe (Lin^-^ c-Kit^+^ Sca-1^-^ CD34^+^ CD16/CD32^hi^ cells) among BM LCs. Cells were pre-gated on the Venus^+^ RFP^+^ Lin^-^ c-Kit^+^ Sca-1^-^ population. (F) Cell cycle distribution of BM LSCe. Cells were pre-gated on the Venus^+^ RFP^+^ Lin^-^ c-Kit^+^ Sca-1^-^ CD34^+^ CD16/CD32^hi^ population. (G) Relative *Notch4* mRNA levels in BM LCs derived from terminally ill, vehicle or AGN193109 treated mice (qRT-PCR). n=3; ****, p<0.0001; t-test. (H) Spleen weights of terminally ill secondary recipients of BM LCs from AGN193109 or vehicle treated, LC^LSK_MA9_shCtrl^ transplanted mice. 2^nd^ BMT, secondary bone marrow transplantation. n = 5; *, p<0.05; t-test.

**Supplementary Figure S5: Effects of the knock-down of *EVI1* on human myeloid cell lines with stem cell characteristics.** HNT-34 and UCSD/AML1 derivative cell lines expressing shEVI1 or control shRen in a doxycycline inducible manner were cultured in the presence or absence of 1 μg/ml doxycycline for 48 h prior to the actual experiments. shRNA expression was leaky, leading to (weaker) phenotypes also in the absence of doxycycline. In some panels, data from Fig. 7 are duplicated to ease comparison between results obtained in the presence or absence of doxycycline. (A, B) Representative immunoblot analyses of HNT-34 (A) and UCSD/AML1 (B) derivative cell lines grown in the presence or absence of doxycycline. (C, D) Expression of the stem cell marker CD34 on HNT-34 (C) and UCSD/AML1 (D) derivative cell lines expressing control or EVI1 shRNAs. Left panels, representative flow cytometric analyses; right panels, mean fluorescence intensities (MFI) of CD34. n=3; **, p<0.01, ns, not significant; ANOVA followed by Bonferroni's post-hoc test. (E-J) Cells were cultured in the presence or absence of 1 μg/ml doxycycline for 48 h, and 1 µM atRA or solvent were added for another 72 h. n=3; *, p<0.05; **, p<0.01; ***, p<0.001; ****, p<0.0001, ns, not significant; ANOVA followed by Bonferroni's post-hoc test. (E, F) Expression of the myeloid differentiation marker CD11b. (E) HNT-34, (F) UCSD/AML1 derivative cell lines. Top panels, representative experiments; bottom panels, quantification. (G, H) Relative viability (measured using metabolic activity as a proxy). (G) HNT-34, (H) UCSD/AML1 cell lines. (I, J) Proportion of quiescent cells (cells in G_0_). (I) HNT-34, (J) UCSD/AML1 derivative cell lines. Top panels, representative experiments, including isotype control for Ki-67; bottom panels, quantification.

**Supplementary Figure S6:** **Effects of atRA and AGN193109 on primary AML samples with high and low *EVI1* expression.** (A) Relative *EVI1* mRNA levels in primary AML samples, determined by qRT-PCR and normalised to β-2-microglobulin expression and to healthy bone marrow sample (hBM) #1. Mean +/- SD from three technical replicates. (B) Primary AML samples were treated with 1 µM atRA, 1 µM AGN193109, or the corresponding amount of solvent (DMSO) for 3 days and subjected to flow cytometric analysis of CD11b expression. Bars represent mean fluorescence intensity (MFI) of CD11b relative to DMSO treated cells. (C) Representative flow cytometric analysis for cell cycle distribution of LSCe (CD34^+^CD38^-^ cells) after three days of treatment with atRA, AGN193109, or solvent. Cells were pre-gated on the CD34^+^CD38^-^ population. The cut-off for Ki-67 negativity was determined using an isotype control antibody (top panels).
