# Supplementary material for: All-trans retinoic acid enhances, and a pan-RAR antagonist counteracts, the stem cell promoting activity of EVI1 in acute myeloid leukemia
Source: Cell Death Dis. 2019 Dec 10;10(12):944. doi: 10.1038/s41419-019-2172-2 (PMC6904467; doi:10.1038/s41419-019-2172-2)
Supplement: Supplementary file 3 — Supplemental Table S1 [file 41419_2019_2172_MOESM3_ESM.docx]

**Supplemental Table S1:** Antibodies used for immunoblot analysis and flow cytometry

| **Method** | **Target** | **Clone/source**  **organism** | **Fluorophor/**  **conjugate** | **Company** | **Dilution** |
| --- | --- | --- | --- | --- | --- |
| IB | Human EVI1 | Rabbit | - | Cell Signaling | 1:1000 |
|  | Human GAPDH | Rabbit | - | Cell Signaling | 1:50,000 |
|  | Flag tag | Mouse | - | Sigma-Aldrich | 1:1000 |
|  | Rabbit IgG | Goat | Horseradish peroxidase | Jackson ImmunoResearch | 1:5000 |
|  | Mouse IgG | Goat | Horseradish peroxidase | Jackson ImmunoResearch | 1:10,000 |
| FC | Mouse Gr-1 | RB6-8C5 | AF700 | Biolegend | 1:100 |
|  | Mouse Gr-1 | RB6-8C5 | APC | Biolegend | 1:100 |
|  | Mouse/human CD11b | M1/70 | AF700 | Biolegend | 1:100 |
|  | Mouse CD3 | 17A2 | AF700 | Biolegend | 1:100 |
|  | Mouse B220 | RA3-6B22 | AF700 | Biolegend | 1:100 |
|  | Mouse Ter119 | TER119 | AF700 | Biolegend | 1:100 |
|  | Mouse c-Kit | 2B8 | PE | Biolegend | 1:50 |
|  | Mouse c-Kit | 2B8 | APC-Cy7 | Biolegend | 1:50 |
|  | Mouse Sca-1 | D7 | BV421 | BD Bioscience | 1:50 |
|  | Mouse Sca-1 | D7 | PerCP/Cy5.5 | Biolegend | 1:50 |
|  | Mouse CD34 | RAM34 | FITC | eBioscience | 1:50 |
|  | Mouse CD34 | MEC14.7 | PE/Cy5.5 | Biolegend | 1:50 |
|  | Mouse CD16/CD32 | 93 | PE/Cy7 | eBioscience | 1:50 |
|  | Mouse Ki-67 | 16A8 | APC | Biolegend | 1:50 |
|  | Human Ki-67 | Ki-67 | APC | Biolegend | 1:50 |
|  | Human CD34 | 581 | AF700 | Biolegend | 1:100 |
|  | Human CD38 | HIT2 | FITC | Biolegend | 1:100 |
|  | Mouse NOTCH4 | HMN4-14 | APC | Biolegend | 1:100 |

IB, immunoblot; FC, flow cytometry. AF700 labelled Gr-1, CD11b, CD3, B220, and Ter119 antibodies were combined to define Lin^-^ cells.
