# Supplementary material for: All-trans retinoic acid enhances, and a pan-RAR antagonist counteracts, the stem cell promoting activity of EVI1 in acute myeloid leukemia
Source: Cell Death Dis. 2019 Dec 10;10(12):944. doi: 10.1038/s41419-019-2172-2 (PMC6904467; doi:10.1038/s41419-019-2172-2)
Supplement: Supplementary file 4 — Supplemental Table S5 [file 41419_2019_2172_MOESM4_ESM.docx]

**Supplemental Table S5:** Clinical characteristics of *de novo* AML patients whose samples were used in this study. BM, bone marrow; PB, peripheral blood; F, female; M, male; Dx, diagnosis; FAB, French American British classification; Hb, hemoglobin; PLT, platelets; WBC, white blood cell count. *EVI1* mRNA levels were determined by qRT-PCR. All samples were from the time of diagnosis. Lack of additional material prohibited testing for recurrent AML associated mutations.

| **Patient No** | **Tissue** | **Sex** | **Age at Dx** | **FAB type** | **Hb [g/L]** | **PLT [G/L]** | **WBC [G/L]** | **Karyotype** | ***EVI1* mRNA level** |
| --- | --- | --- | --- | --- | --- | --- | --- | --- | --- |
| AML #1 | PB | F | 82 | M4 | 89 | 54 | 29,2 | 45,XX,iso1p,-7 | high |
| AML #2 | BM | F | 51 | M4 | 66 | 43 | 16,3 | 46,XX | high |
| AML #3 | BM | M | 72 | M1 | 86 | 190 | 29,6 | 46,XY | high |
| AML #4 | BM | M | 55 | M2 | 65 | 41 | 2,3 | 45,XY,t(6;17) | high |
| AML #5 | BM | F | 37 | M2 | 73 | 41 | 3,3 | 46,XX | low |
| AML #6 | BM | M | 35 | M5 | 50 | 11 | 129,0 | 46,XY | low |
