# Supplementary figures and images for: All-trans retinoic acid enhances, and a pan-RAR antagonist counteracts, the stem cell promoting activity of EVI1 in acute myeloid leukemia
Source: Cell Death Dis. 2019 Dec 10;10(12):944. doi: 10.1038/s41419-019-2172-2 (PMC6904467; doi:10.1038/s41419-019-2172-2)

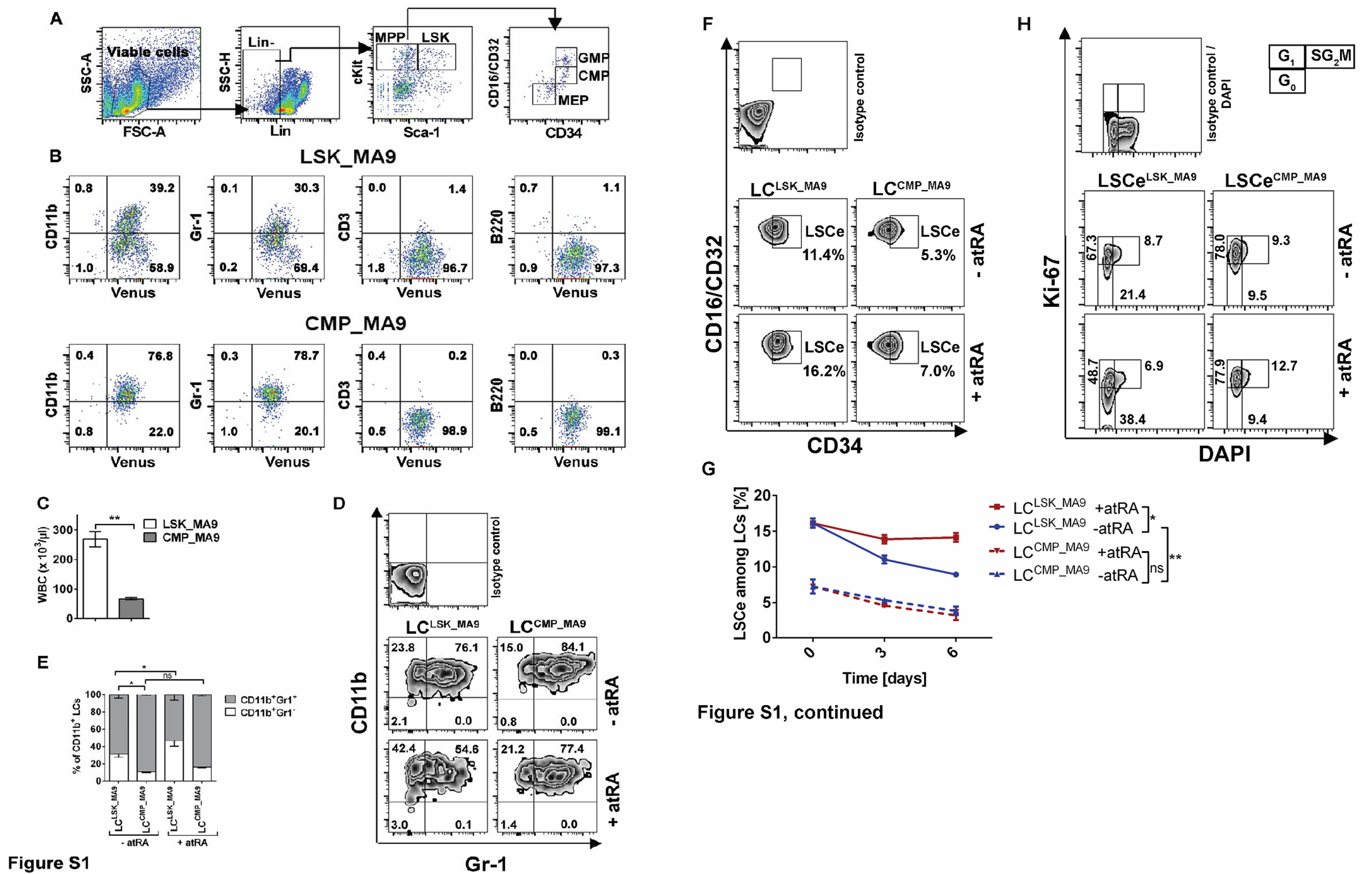

Supplement: Supplementary file 5 — Supplemental Figure S1 [file 41419_2019_2172_MOESM5_ESM.tif]

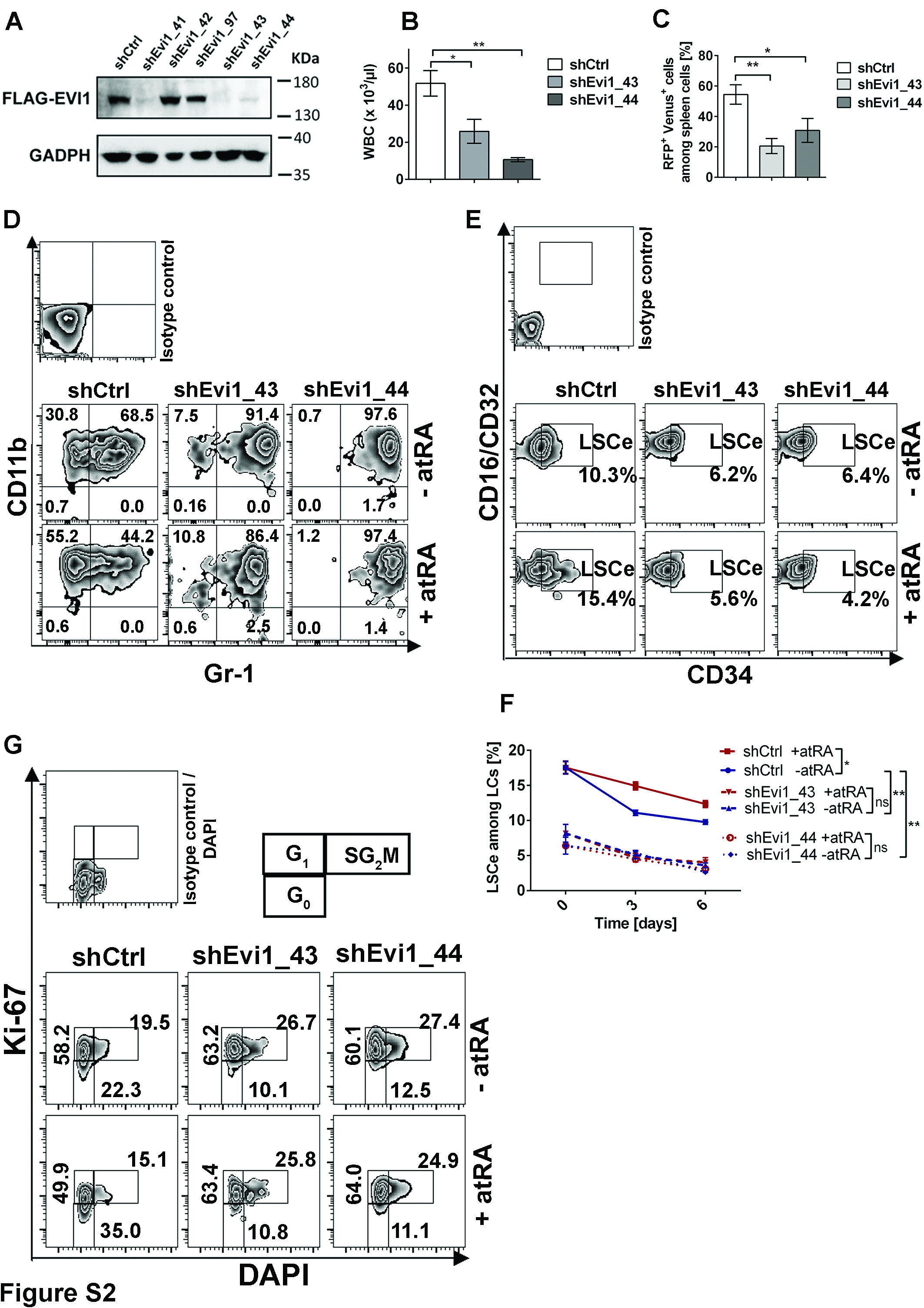

Supplement: Supplementary file 6 — Supplemental Figure S2 [file 41419_2019_2172_MOESM6_ESM.tif]

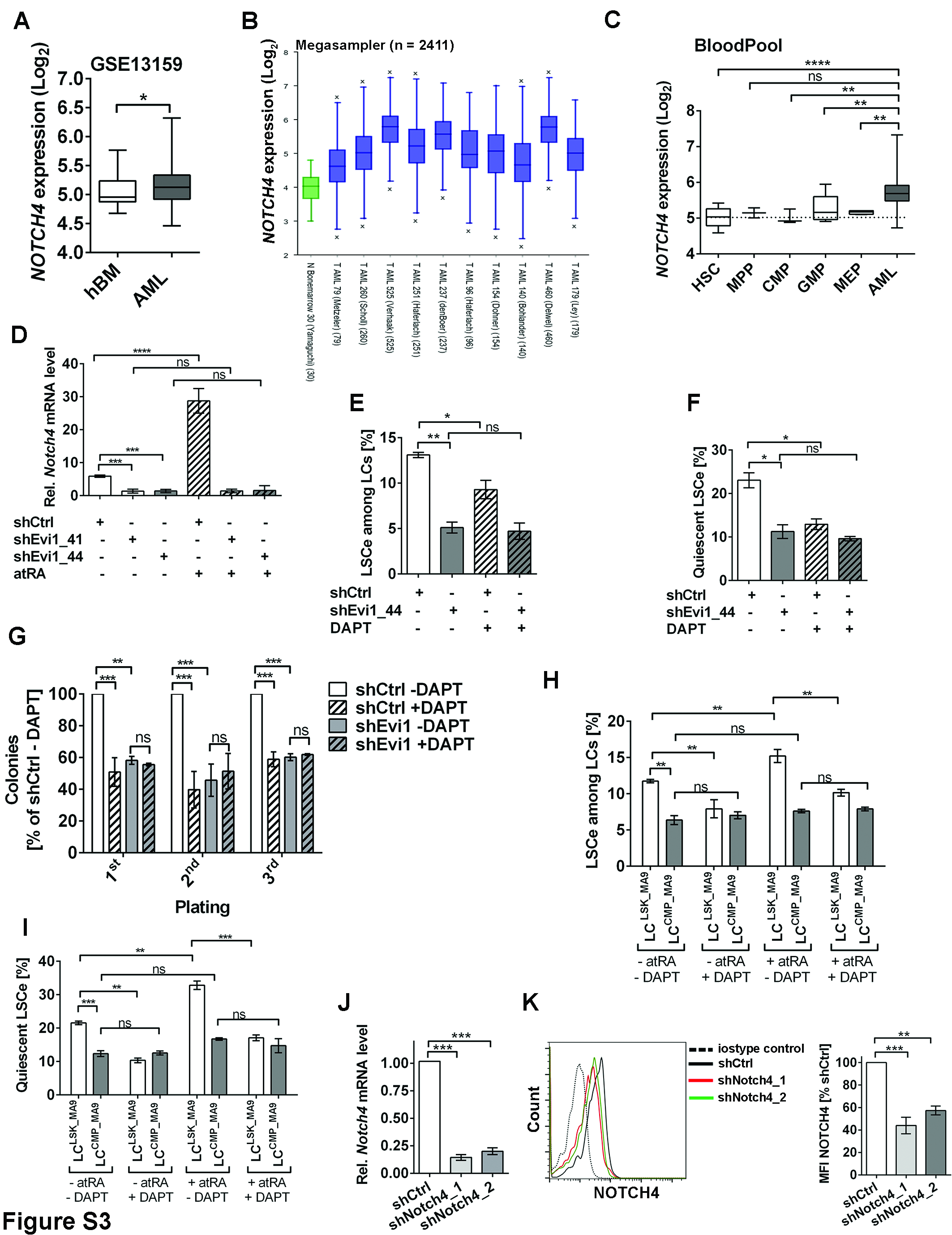

Supplement: Supplementary file 7 — Supplemental Figure S3 [file 41419_2019_2172_MOESM7_ESM.tif]

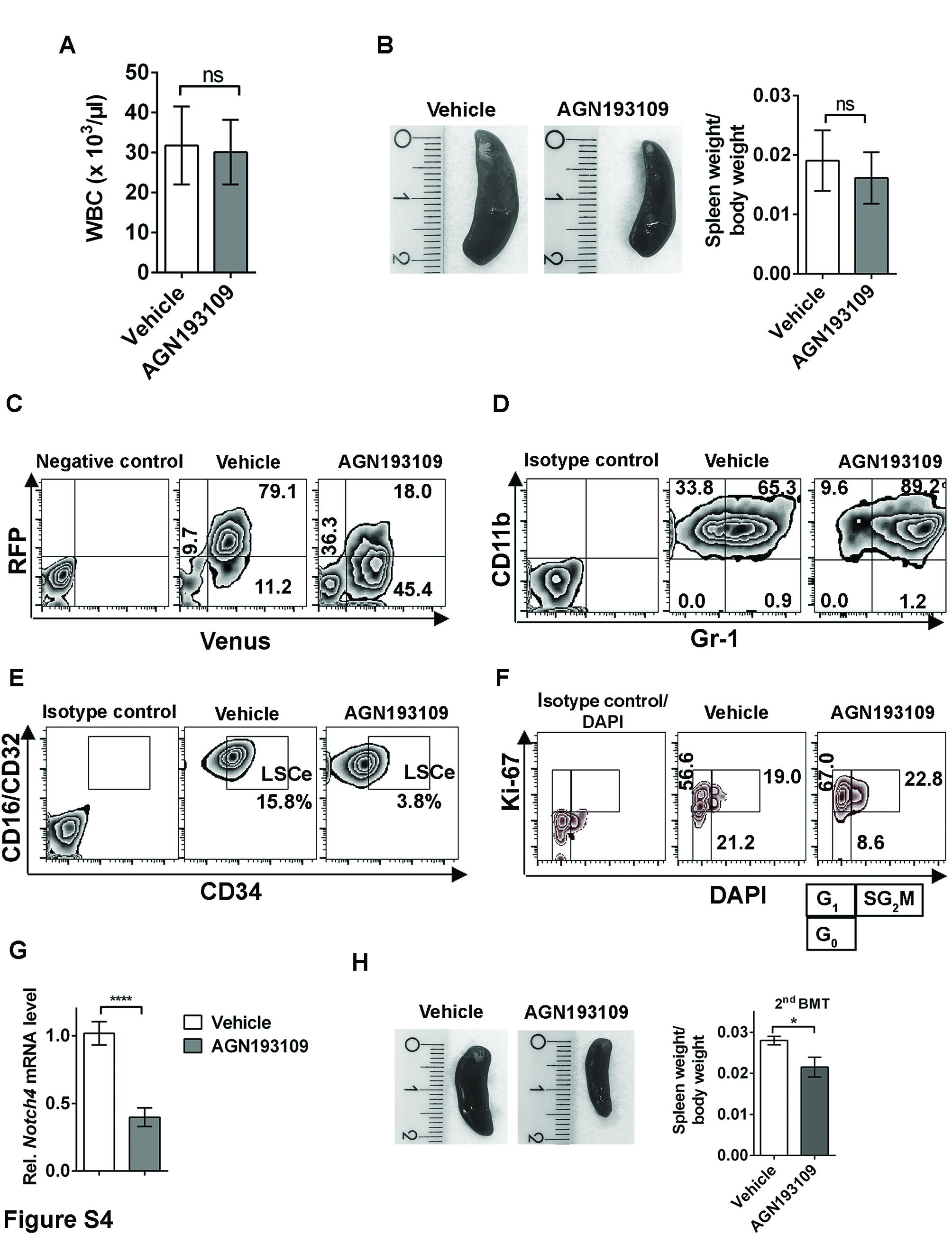

Supplement: Supplementary file 8 — Supplemental Figure S4 [file 41419_2019_2172_MOESM8_ESM.tif]

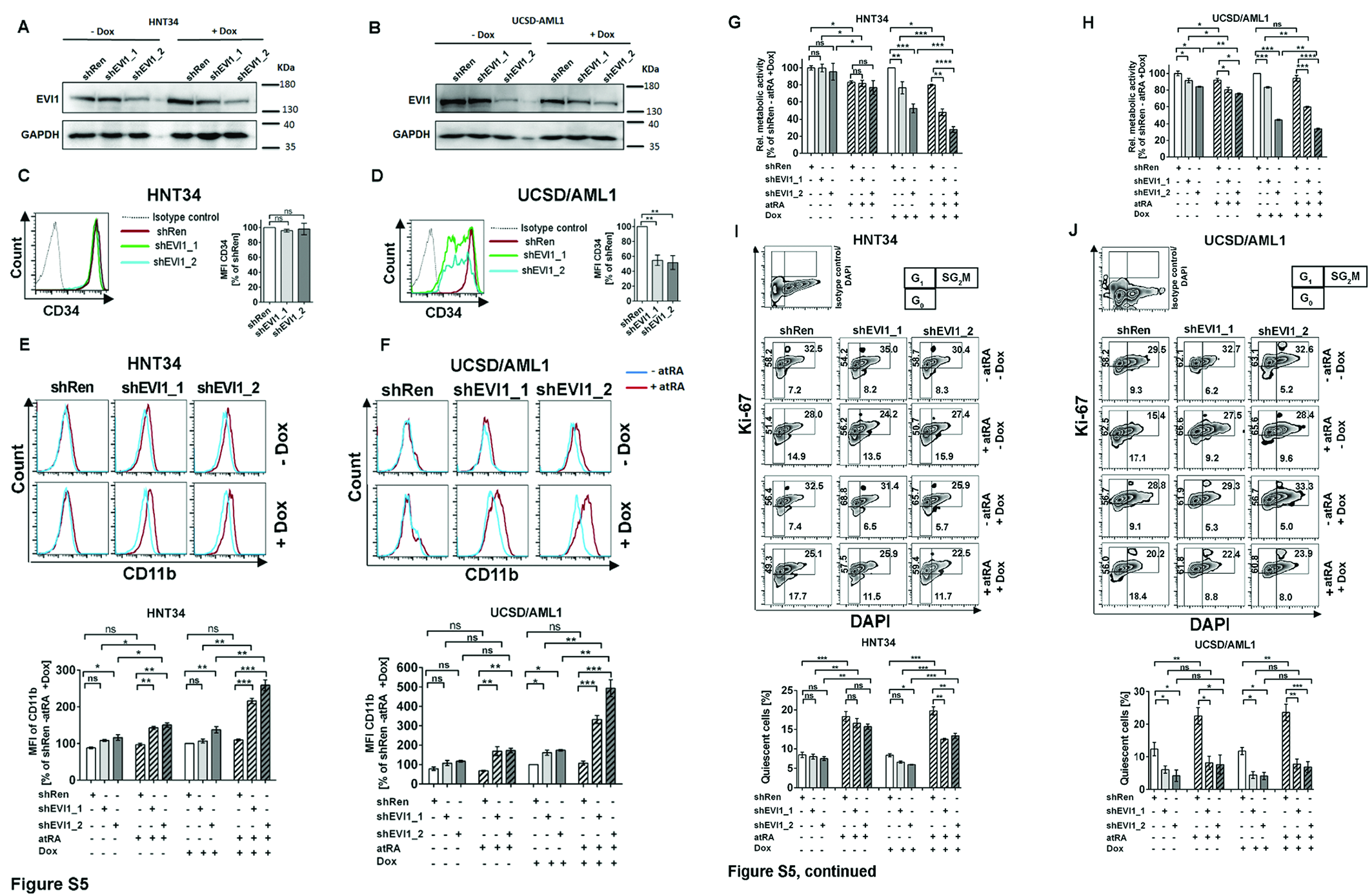

Supplement: Supplementary file 9 — Supplemental Figure S5 [file 41419_2019_2172_MOESM9_ESM.tif]

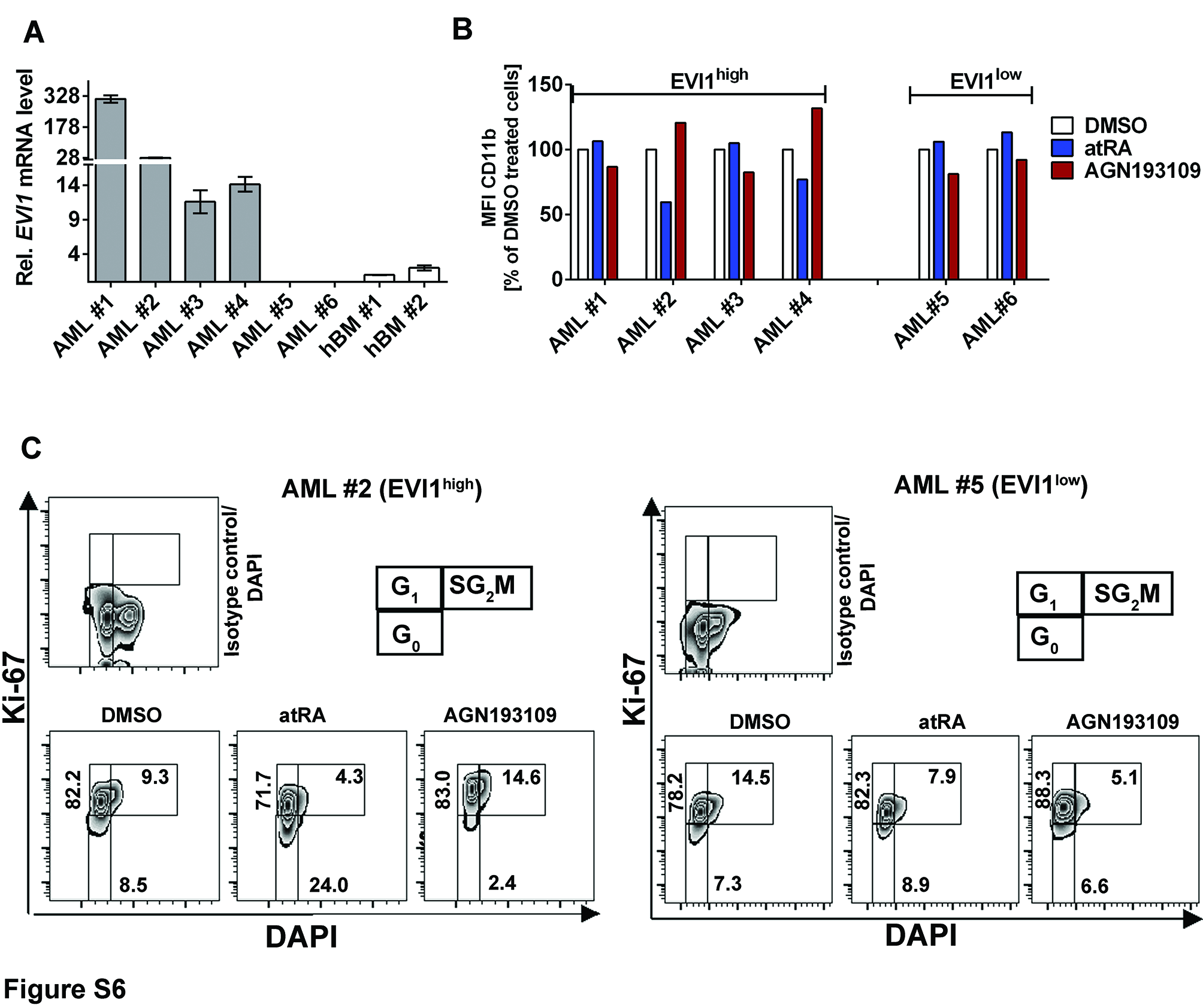

Supplement: Supplementary file 10 — Supplemental Figure S6 [file 41419_2019_2172_MOESM10_ESM.tif]
